# The Significance of Tumor Microenvironment Score for Breast Cancer Patients

**DOI:** 10.1155/2022/5673810

**Published:** 2022-04-28

**Authors:** Yuan Tian, Jingnan Wang, Qing Wen, Aiqin Gao, Alan Huang, Ran Li, Ye Zhang, Guohai Su, Yuping Sun

**Affiliations:** ^1^Department of Oncology, Jinan Central Hospital Affiliated to Shandong University, Jinan, 250013 Shandong, China; ^2^Somatic Radiotherapy Department, Shandong Second Provincial General Hospital, Shandong Provincial ENT Hospital, Jinan, Shandong 250023, China; ^3^State Key Laboratory of Molecular Oncology and Department of Radiation Oncology, National Cancer Center/Cancer Hospital, Chinese Academy of Medical Sciences (CAMS) and Peking Union Medical College (PUMC), Beijing, China; ^4^Jinan Clinical Research Center of Shandong First Medical University, Jinan, China; ^5^Department of Oncology, Jinan Central Hospital, The Hospital Affiliated with Shandong First Medical University, Jinan, Shandong 250013, China; ^6^Department of Oncology, Jinan Central Hospital, Weifang Medical University, Weifang, 261053 Shandong, China; ^7^Department of Cardiovascular Diseases, Jinan Central Hospital Affiliated to Shandong University, Jinan, 250013 Shandong, China; ^8^Phase I Clinical Trial Center, Shandong Cancer Hospital and Institute, Shandong First Medical University and Shandong Academy of Medical Sciences, Jinan, Shandong 250012, China

## Abstract

**Purpose:**

This study was designed to clarify the prognostic value of tumor microenvironment score and abnormal genomic alterations in TME for breast cancer patients.

**Method:**

The TCGA-BRCA data were downloaded from TCGA and analyzed with R software. The results from analyses were further validated using the dataset from GSE96058, GSE124647, and GSE25066.

**Results:**

After analyzing the TCGA data and verifying it with the GEO data, we developed a TMEscore model based on the TME infiltration pattern and validated it in 3273 breast cancer patients. The results suggested that our TMEscore model has high prognostic value. TME features with the TMEscore model can help to predict breast cancer patients' response to immunotherapy and provide new strategies for breast cancer treatment. Signature 24 was first found in breast cancer. In focal SCNAs, a total of 95 amplified genes and 169 deletion genes in the TMEscore high group were found to be significantly related to the prognosis of breast cancer patients, while 61 amplified genes and 174 deletion genes in the TMEscore low group were identified. LRRC48, CFAP69, and cg25726128 were first discovered and reported to be related to the survival of breast cancer patients. We identified specific mutation signatures that correlate with TMEscore and prognosis.

**Conclusion:**

TMEscore model has high predictive value regarding prognosis and patients' response to immunotherapy. Signature 24 was first found in breast cancer. Specific mutation signatures that correlate with TMEscore and prognosis might be used for providing additional indicators for disease evaluation.

## 1. Introduction

The ever-increasing wealth of accessible sequencing data has enabled deeper analysis of the genetic alterations underlying cancer [1–8], and reanalysis of the existing online data has become a common method for cancer research [9, 10]. The tumor microenvironment (TME) [11, 12], especially the immune tumor microenvironment (iTME) [13], has been reported to play an important role in tumor progression, metastasis, prognosis, and immunotherapies [14]. Reanalysis of existing tumor samples and TME datasets has increased our knowledge of intercellular interactions and genomic abnormalities in tumorigenesis, enabling researchers to identify effective targets for clinical treatment [10, 15, 16]. In iTME, the diagnosed TME context was associated with immune response and chemotherapy benefits [17, 18]. Furthermore, alteration of immune-related indicators, such as CD4+, CD8+, macrophage, and infiltrating levels, has a significant impact on the prognosis of various tumors including breast cancer [19–22].

Through the analysis of immune cell interactions, genome, and transcriptome alterations in the breast cancer microenvironment, it also suggested that abnormal alterations in TME have important effects on the occurrence, development, metastasis, immunotherapy, and prognosis [23–27]. In order to better understand abnormal alterations in TME among breast cancer patients, we reanalyzed published datasets to deconvolve (CIBERSORT) and determine the TME profile of breast cancer samples [28]. Moreover, we correlated the TME phenotypes with genomic characteristics and pathologic features of breast cancer, thereby providing a new model for breast cancer prognosis.

## 2. Materials and Methods

### 2.1. Published Datasets

Datasets GSE96058, GSE124647, and GSE25066 were downloaded from GEO (https://www.ncbi.nlm.nih.gov/geo/), while the data of TCGA-BRCA (RNA-seq, miRNA expression data, methylation, and clinical data) were downloaded from the UCSC Xena browser (https://xenabrowser.net/datapages/). Mutation data of TCGA-BRCA were downloaded by R TCGAbiolinks. The raw data related to Affymetrix were checked for background adjustment in the Affy software package by the RMA algorithm [29]. The raw data related to Illumina were processed by the Lumi software package.

After removing duplicate data, 3273 samples of GSE96058 with information of RNA-seq expression and clinical data were obtained and used for downstream analysis. Data of GSE124647 (140 samples) and GSE25066 (508 samples) were used for validation. Genome-Wide Human SNP 6.0 copy number segment data of TCGA-BRCA were downloaded from Firebrowse (http://firebrowse.org/) and processed using GISTIC 2.0. After removing duplicated data and samples without survival information, 1057 transcriptome samples were validated for TMEscore [10]. Among these 1057 samples, 1039 samples had CNV data, 1042 samples had SNP data, 1038 samples had miRNA data, and 760 samples had methylation data. The basic characteristics of the above 4 datasets are displayed in (Supplementary Table 1).

### 2.2. Comprehensive Analysis for TME

#### 2.2.1. Proportion of Infiltrating Cell Evaluation and TME Cluster Identification

The CIBERSORT algorithm and leukocyte signature matrix (LM22) gene signature were used for calculating immune cell infiltration of 22 human immune cell phenotypes [28, 30]. CIBERSORT was considered as a deconvolution algorithm that used a set of gene expression values to calculate a minimal representation for each cell type [30]. Based on these values, support vector regression could be used to infer the cell type ratio for data from large tumor samples with mixed cell types [30]. We used standard annotation files to prepare gene expression profiles and uploaded the data to the CIBERSORT web portal (http://cibersort.stanford.edu/). Then, we used LM22 signatures and 1,000 permutations to run the algorithm. By applying the microenvironmental cell population counting method to evaluate the proportion of stromal cells, the absolute abundance of eight immune cells and two stromal cell populations in heterogeneous tissues from transcriptome data was quantitatively analyzed [10, 30].

#### 2.2.2. Unsupervised Clustering to Identify TME Patterns and Tumor Sample Classification

**Figure 1:**
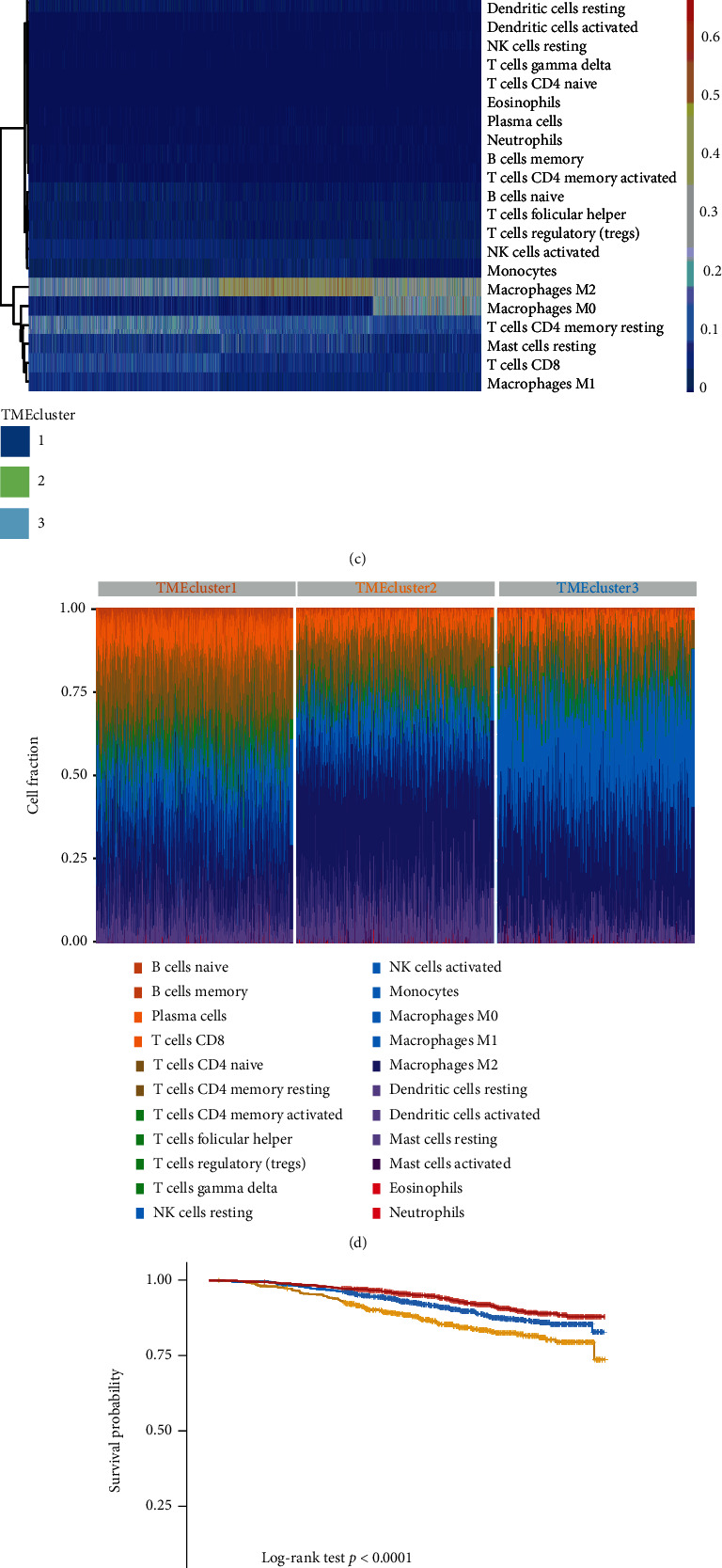
The pattern classification of tumor microenvironment (TME). (a) Optimal number of clusters: *K* value calculated by the elbow method and gap statics algorithm. The ordinate axis represents total within sum of square; the abscissa axis represents the number of clusters *K*. (b) Consensus matrix heat map (*K* = 3): ConsensusClusterPlus was used for unsupervised class discovery (1000 iterations, *k* = 1 : 10). The optimal *k* value of 3 was determined using the elbow method and gap statics, combined with the correlation between the final classification and survival. (c) The distribution ratio of all kinds of immune cells in different TME clusters. (d) Clustering heat map of the distribution ratio of all kinds of immune cells in different TME clusters. (e) Survival analysis for different TME clusters: the red curve represents the TME cluster 1, the blue curve represents the TME cluster 2, and the yellow curve represents the TME cluster 3. The ordinate axis represents the probability of survival, and the abscissa axis represents the survival days.

From the immune cell proportion data analyzed by CIBERSORT [28, 30], elbow (the error of the squares sum within the WSSE group, this method was used to find the optimal number of clusters by finding the “elbow point”) (Figure 1(a)) and gap statics (the point where *k* drops fastest the *k* value corresponding to the maximum gap value) were applied to evaluate the best number of the categories *k* value, and ConsensusClusterPlus R package was used for classification to obtain TME cluster (k-means, Euclidean, and ward.D) [31–33]. In order to obtain stable classification, the above procedure was repeated for 1000 times. Then, TME clusters were combined with survival data to clarify whether this classification was related to survival (Figures 1(b) and 1(c)).

#### 2.2.3. TME Scoring according to the Differentially Expressed Genes (DEG) among TME Clusters

Based on the above results, different TME clusters were mapped to the RNA-seq data and screened for DEG in different samples by R limma package (*P* < 0.05 and |log_2_FC| > log_2_ (1.5)) [34]. The Benjamini–Hochberg correction was used to adjust *P* value for multiple testing [35]. After selecting category-specific differential genes, redundant genes were removed by random forest method [36]. Signature genes were obtained and functional enrichment analysis was applied to annotate the mainly enriched pathways. Genes were divided into two categories by the Cox regression model according to the coefficient values (positive or negative). Referring to gene expression grade index (GGI) score, TMEscore was calculated by the following formula [37]:

(1) $$\begin{array}{c} \text{TMEscore}=\sum \log_{2}\left(X+1\right)-\sum \log_{2}\left(Y+1\right), \end{array}$$

where *X* is the positive expression value of the Cox coefficient responding to a gene set and *Y* is the expression value of the Cox coefficient involving gene set. The samples were then divided into TMEscore high and TMEscore low groups based on the median.

Based on the above results, other data, such as TCGA and GEO, could be applied to verify this model. Then, TMEscore would be divided into TMEscore high and low groups according to the median, and the correlation between the two groups and prognosis would be analyzed. In the end, the correlation between the top 10 differentially expressed genes (DEG) and the prognosis was validated by online database analysis, which would be displayed as the Kaplan-Meier curves.

### 2.3. Analysis of TCGA-BRCA Mutation Spectrum

#### 2.3.1. SNP-Related Mutation Spectrum Analysis

By taking intersections between the mutation data and RNA-seq data from TCGA-BRCA, 1042 samples were obtained to analyze the SNP spectrum using R Maftools (https://bioconductor.org/packages/release/bioc/html/maftools.html) and Somatic Signatures packages (https://bioconductor.org/packages/release/bioc/html/SomaticSignatures.html). The Spearman rank correlation was utilized to evaluate the relationship between TMEscore and mutation load of tumor subtype. Pancancer survival analysis of mutational genes was put into practice by starbase online (http://starbase.sysu.edu.cn/panGeneSurvivalExp.php).

#### 2.3.2. Copy Number Variant (CNV) Analysis

According to the intersection of the processed SNP6 copy number segment data of breast cancer samples downloaded from the website (http://firebrowse.org/) and the RNA-seq data samples used above, 1,039 samples were obtained. The common CNV area in all samples, including the chromosome arm-level CNV and the smallest common area between samples, were detected by the Genomic Identification of Significant Targets in Cancer (GISTIC) method according to the SNP6 copy number segment data. The parameters of the GISTIC method were set as follows: *Q* ≤ 0.05 is considered as a significant alteration standard; a confidence level of 0.95 was used to determine the peak interval; the area range greater than 0.98 of the chromosome arm length was used as the standard for analyzing the chromosome arm-level variation. The above analyses were performed through the corresponding MutSigCV module in the online analysis tool Gene Pattern (https://cloud.genepattern.org/gp/pages/index.jsf) developed by Broad Research Institute.

#### 2.3.3. Tumor Purity and Ploidy Analysis

Tumor purity and ploidy analysis were carried out by R “ABSOLUTE” package (https://software.broadinstitute.org/cancer/cga/absolute_download). By leveraging copy number and mutation data, ABSOLUTE was able to estimate the purity and ploidy of samples.

### 2.4. Comprehensive Analysis

#### 2.4.1. TME and Gene Expression Correlation Analysis

Gene expression profile (mRNA and miRNA) was used to identify genes specifically expressed in different subgroups. Later, functional enrichment analysis of specifically expressed genes was performed to elucidate the differences in the biological functions of different TME subgroups [20].

#### 2.4.2. Prognostic Evaluation of TME Cluster and TMEscore

The differentially expressed genes (DEG), miRNAs, and methylation sites were firstly identified between TMEscore subgroups (TMEscore high vs. low). After combining clinical data, survival analysis of these DEG, miRNAs, and methylation sites was further performed to determine whether they were related to clinical outcomes [34]. The molecular and clinical characteristics of different TME model subgroups were described through multidimensional data, and then, they were used for constructing the landscape map of the studied tumor.

#### 2.4.3. Exploring the Relationship between TMEscore and the Prognosis of Immune Checkpoint Inhibitor (ICI) Treatment

TMB is a feature that is known to be significantly related to immunotherapy and can be used to predict the efficacy of immunotherapy [38]. ROC was used to evaluate the predictability of TMB, TME group, and TMB+TME group regarding the effect of immunotherapy. The prognostic significance of the TME cluster and TMEscore was evaluated by the Kaplan-Meier curves and Cox proportional hazard regression models. The prognostic value of the TMEscore was further validated in several datasets with different biological or therapeutic background, including TCGA-BRCA, GSE124647 (metastatic breast cancer receiving endocrine therapy), and GSE25066 (patients receiving neoadjuvant taxane-anthracycline chemotherapy). TIDE (http://tide.dfci.harvard.edu/) was used for evaluating the clinical effects of ICI therapy whereby a higher tumor TIDE score was related to a poorer responsiveness to ICI and prognosis.

## 3. Results

### 3.1. The Breast Cancer TME Landscape

We used CIBERSORT to estimate the abundances of 22 immune cell types (memory B cells, activated dendritic cells, M0 macrophages, etc.) in 3273 different breast cancer RNA-seq datasets (S Figure 1A) and correlated the immune cell profile with survival (S Figure 1B). The basic information of 22 kinds of immune cells in 3273 samples are provided in Supplementary table 1. Next, we used ConsensusClusterPlus for unsupervised class discovery (1000 iterations, *k* = 1 : 10). The optimal *k* value of 3 was determined using the elbow method and gap statics, combined with the correlation between the final classification and survival (Figures 1(a)–1(e) and Methods).

**Figure 2:**
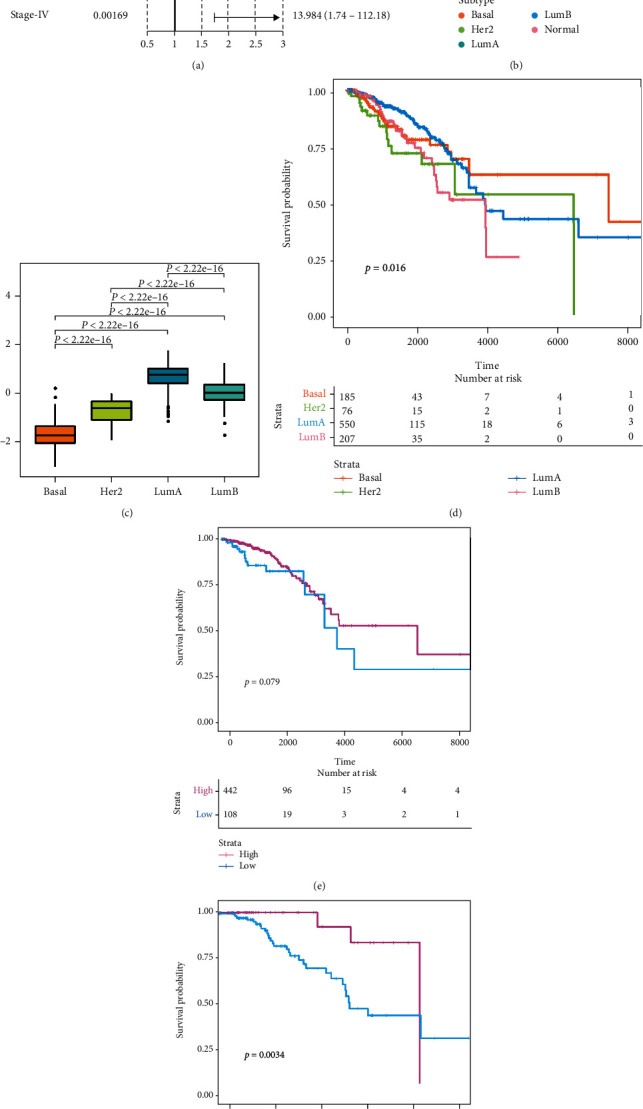
Evaluation of TMEscore model and analysis of its correlation with mutation load. (a) The meta-analysis results of TMEscore model in different datasets: training set, testing set, TCGA, TCGA different stages, metastatic breast cancer, and prognosis evaluation of drug treatment. (b) Correlation analysis between TMEscore and mutation loads in different subtypes (basal, Her 2, Lum A, Lum B, and normal): the ordinate axis represents total mutations, and the abscissa axis represents TMEscore. (c) TMEscore box plots in different subtypes (basal, Her 2, Lum A, and Lum B). (d) Survival analysis results in four different subtypes of breast cancer (basal, Her 2, Lum A, and Lum B): the ordinate axis represents the probability of survival, and the abscissa axis represents the survival days. Different colors represent different subtypes. (e) Survival analysis of luminal A subtype after grouping according to TMEscore: the ordinate axis represents the probability of survival, and the abscissa axis represents the survival days. Different colors represent different TMEscore subgroups (high TMEscore and low). (f) Survival analysis of luminal B subtype after grouping according to TMEscore: the ordinate axis represents the probability of survival, and the abscissa axis represents the survival days. Different colors represent different TMEscore subgroups (high TMEscore and low).

According to the above TME classification (*k* = 3; Figure 1(b)), we performed gene expression analysis using *limma* and identified 552 differentially expressed genes (DEGs) (*P* < 0.05, |log_2_FC| > log_2_ (1.5)). Unsupervised clustering then classified the differentially expressed genes into three groups (S Figure 2A). Functional enrichment analysis on 177 nonredundant genes using R ClusterProfiler revealed that this gene set was significantly enriched in immune-related pathways such as lymphocyte migration, lymphocyte chemotaxis, and leukocyte chemotaxis (S Figure 2B). A Cox regression model was used to determine the relationship between DEGs and the survival of samples. Next, genes were divided into 2 categories according to their coefficient values, and samples were divided into two groups based on calculated high or low TMEscores.

We found that the samples in the high TMEscore group had a good prognosis, while samples in the low TMEscore group had a poor prognosis (S Figure 2C). This finding indicated that clustering samples based on their immune cell profile combined with a TMEscore could predict the prognosis of breast cancer patients.

**Figure 3:**
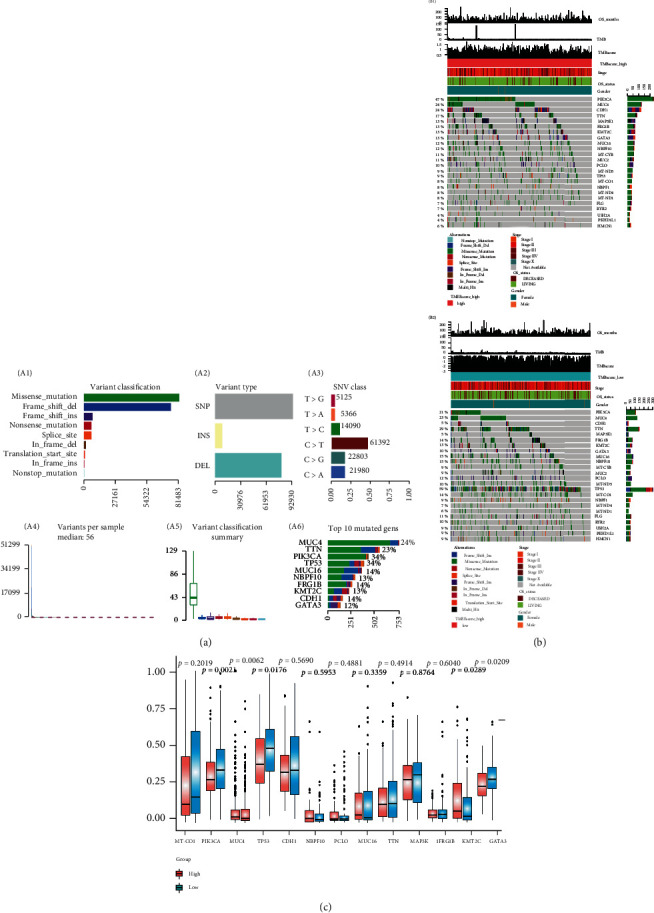
Overview of mutations in 1039 TCGA-BRCA samples. (a) Tumor mutation profile. A1: variant classification of 1039 tumor samples. Missense mutations were the main mutation type in BRCA. A2: variant type of 1039 tumor samples. The source of mutations was mainly SNPs (mostly C>T) followed by indels. A3: SNV class of 1039 tumor samples. A4: variants per sample among 1039 tumor samples. A5: variant classification summary of 1039 tumor samples. A6: top 10 mutated genes in 1039 tumor samples. MUC4 was the most common mutated gene, followed by TTN. (b) Gene mutation distribution and phenotype in different TMEscore groups. B1: the distribution of mutations and mutation annotations of 24 genes in TMEscore high group. B2: the distribution of mutations and mutation annotations of 24 genes in TMEscore low group. (c) The frequency distribution of common gene mutations. Among them, the mutation rates of PIK3CA, TP53, KMT2C, GATA3, and MUC4 in the two subgroups have statistically significant differences.

We next evaluated the TMEscore model using datasets representing metastatic breast cancer (TCGA-BRCA), breast cancer receiving endocrine therapy (GSE124647), and breast cancer receiving neoadjuvant taxane-anthracycline chemotherapy (GSE25066) (S Figure 3A-D). We found that the calculated TMEscore could effectively predict the prognosis in these samples. Namely, there is a significant negative correlation between TMEscore and mutational load of metastatic breast cancer samples (Spearman coefficient *R* = −0.44, *P* < 2.2 × 10^−16^) (Figures 2(a) and 2(b)). TCGA-BRCA was grouped and evaluated according to luminal A, luminal B, basal, and Her-2. The TMEscores between different subtypes were significantly different. The TMEscores of basal and Her 2 were significantly lower than the others, and luminal B was the second highest, while luminal A was the highest (Figure 2(c)). The overall survival cure is displayed in (Figure 2(d)); the stratified analysis results are shown in (Figures 2(e) and 2(f)).

### 3.2. Breast Cancer Mutational Analysis

#### 3.2.1. Overview of Mutations

We performed statistical analysis on the mutational data of 1039 tumor samples (TCGA-BRCA), including the type of mutation annotation, the proportion of different types of base changes, and top 10 mutation genes (Figure 3(a)). Missense mutation was the major mutation type in BRCA, and the major source of mutations was SNPs (mostly C>T), followed by indels. In these tumor samples, the top 10 mutated genes included PIK3CA, MUC4, and TTN (Figures 3(b) and 3(c)). According to the tumor mutation burden (TMB) score's high/low groups of BRCA samples, the distribution of mutations and mutation annotations of 24 (the union of the top 20 of each mutation) genes are listed in Figures 3 B1 and 3 B2. The frequency distribution of common gene mutations is shown in Figure 3(c).

#### 3.2.2. Mutation Spectrum Analysis

**Figure 4:**
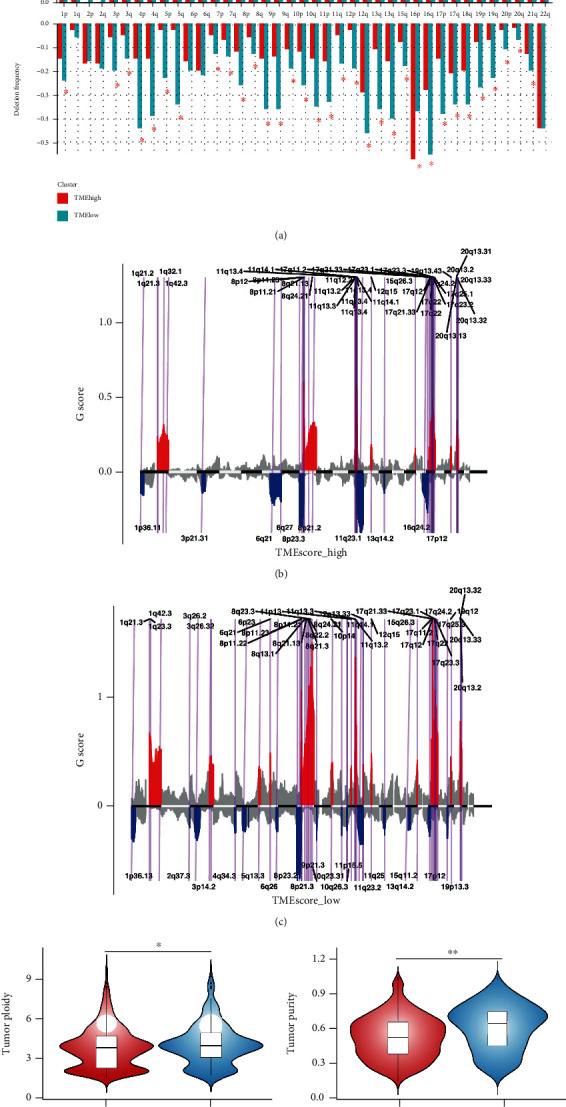
The analysis results of CNV. (a) The occurrence of chromosome arm-level amplification and deletion in different TMEscore groups: the abscissa axis represents the chromosome locus, and the ordinate axis represents the frequency of copy number alterations. Red represents the high TMEscore group, and the other one represents the low TMEscore group. ∗ represents statistical differences in frequency between the two groups. (b) Distribution of copy number amplification and deletion regions in high TMEscore group: 11q13.3 was the most significant in the amplified region, and 11q23.1 was the most significant in the deletion region. (c) Distribution of copy number amplification and deletion regions in TMEscore_low group: the most significant amplification region was located at 8q24.21, and the most significant deletion region was located at 8p23.2. (d) Ploidy analysis results in high and low TMEscore groups: ∗ represents statistical differences in frequency between the two groups. (e) Purity analysis results in high and low TMEscore groups: ∗∗ represents statistical differences in frequency between the two groups.

By annotating the bases immediately upstream or downstream each mutation site, we identified 96 mutation contexts and counted their frequencies in the BRCA tumor samples (S Figure 4A and 4B). In order to determine the relationship between the mutation frequency distribution of BRCA tumor samples and the signature included in COSMIC, we performed nonnegative matrix decomposition of the frequency matrix with 1042 samples in rows and 96 mutation types in columns. After extracting mutational characteristics of 3 somatic point mutations, the similarity between the extracted features and the signature collected by cosmic was analyzed. Through analysis, we found that the mutational spectrum of the high TMEscore group was mainly related to Signature 2, Signature 10, and Signature 30 (S Figure 4C), while the low TMEscore group was mainly related to Signature 2, Signature 3, and Signature 6 (S Figure 4D).

#### 3.2.3. Analysis of Copy Number Variation (CNV)

Two sets of BRCA samples were analyzed by GISTIC software. In the high TMEscore group, 1q allelic amplification and 16q allelic deletion were the most significant alterations (Figure 4(a)), while 1q allelic amplification and 17p allelic deletion were the most significant alterations in the low TMEscore group.

Additionally, a total of 41 amplifications and 12 copy number deletions were detected among tumor samples in the high TMEscore group (Figure 4(b)). Among them, 11q13.3 was the most significant in the amplified region, while 11q23.1 was the most significant in the deletion region. In the low TMEscore group, 41 amplification and 24 deletions were found. The most significant amplification region was located at 8q24.21; and the most significant deletion region was located at 8p23.2 (Figure 4(c)).

**Figure 5:**
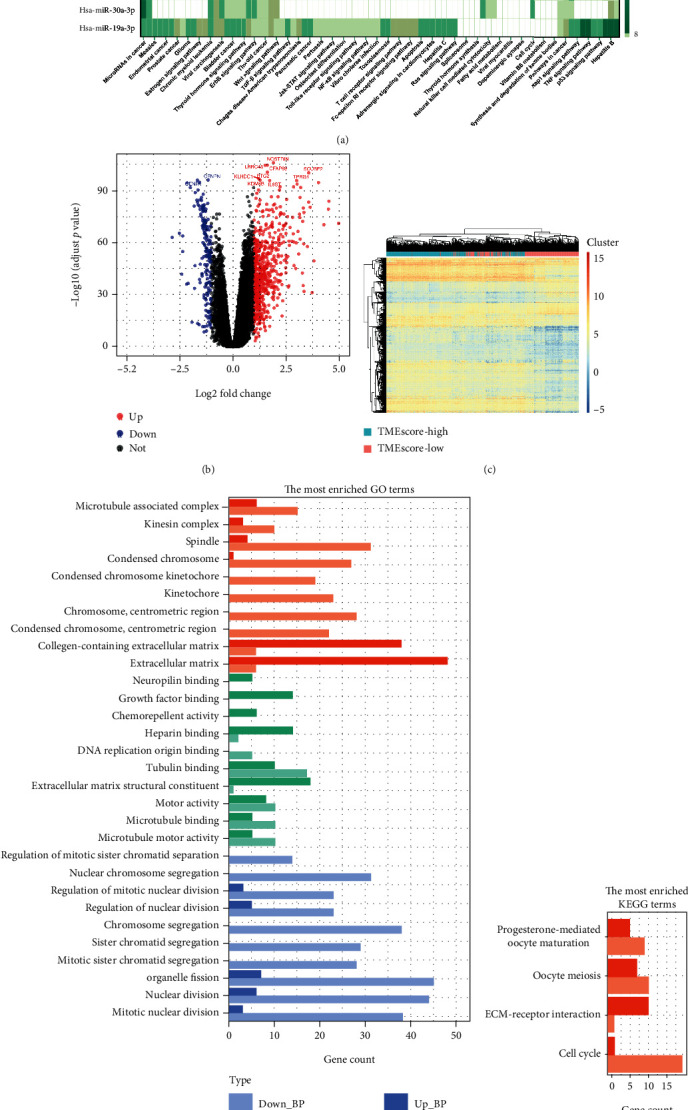
The analysis results of miRNA and mRNA. (a) Functional annotation of differentially expressed miRNA: the ordinate axis represents different miRNAs, and the abscissa axis represents the functional annotations of miRNAs. (b) Volcano map of differentially expressed genes (DEG): the red part on the right shows the upregulated TOP 9 genes, and the blue on the left shows the downregulated top 2 genes. (c) Heat map of differentially expressed genes (DEG). (d) GO enrichment analysis of differentially expressed genes (DEG): the abscissa axis represents the number of genes, and the ordinate axis represents the results of CC (cellular component), BP (biological process), and MF (molecular function). (e) KEGG enrichment analysis of differentially expressed genes (DEG): the abscissa axis represents the number of genes, and the ordinate axis represents the corresponding pathways.

We then applied Pancancer survival analysis of genes across 32 types of cancers in both high and low TMEscore groups (Methods http://starbase.sysu.edu.cn/panGeneSurvivalExp.php). A total of 95 amplified genes and 169 deletion genes in the high TMEscore group and while 61 amplified genes and 174 deletion genes in the low TMEscore group were found to be significantly related to the prognosis of breast cancer patients (Tables 1 and 2; S Figure 5). In the high TMEscore group, the chromosomal locus with the highest number of amplified genes was located at 8q24.21 (*n* = 16; S Figure 5A1), and the highest number of deleted genes was located at 1p36.11 (*n* = 52; S Figure 5A2). However, in the low TMEscore group, the chromosomal locus with the highest number of amplified genes was found at 10p14 (*n* = 11, S Figure 5B1), and the highest number of deleted genes was found at 1p36.13 and 3p14.2 (*n* = 36; S Figure 5B2).

Based on the CNV information of each tumor sample, the tumor purity and ploidy was evaluated by ABSOLUTE software (Figure 4(e)). Tumor purity ranged from 0.16 to 1, and tumor cell genome ploidy ranged from 1.54 to 9.79, suggesting that genome disorder was common during tumorigenesis. Moreover, we noted a significant difference in the ploidy of the tumors in the high/low TMEscore groups (*t*-test, *P* = 2.337e − 08, and *P* = 0.03075; Figure 4(d)).

### 3.3. miRNA, mRNA, and Methylation Analysis

#### 3.3.1. miRNA Differential Expression Analysis

Samples with high or low TMEscores were further analyzed using *limma* to identify differentially expressed miRNAs (adj.*P* < 0.05, |log2FC| > 1), followed by functional annotation (Methods). We detected 29 differentially expressed miRNAs, including hsa-miR-19a-3p, that were related to cancer and immune pathways (Figure 5(a)). Importantly, hsa-mir-19a-3p, hsa-mir-30a-3p, and hsa-mir-9-5p have been validated to be significantly correlated with the survival of breast cancer patients (http://starbase.sysu.edu.cn/panMirSurvivalExp.php#).

#### 3.3.2. mRNA Differential Expression Analysis

Gene expression analysis comparing samples with high and low TMEscores (adj.*P* < 0.05, |log2FC| > 1) followed by functional annotation (Methods) identified 782 differentially expressed genes (DEGs). Notably, DEGs were mainly upregulated in the samples with high TMEscores (Figures 5(b) and 5(c)). Most DEGs were enriched in the cell cycle, cell division, and other related pathways, indicating that the major biological differences between two sets of samples lie in cell proliferation (Figures 5(d) and 5(e)).

#### 3.3.3. Analysis of Differences in the Expression of Methylation Sites

**Figure 6:**
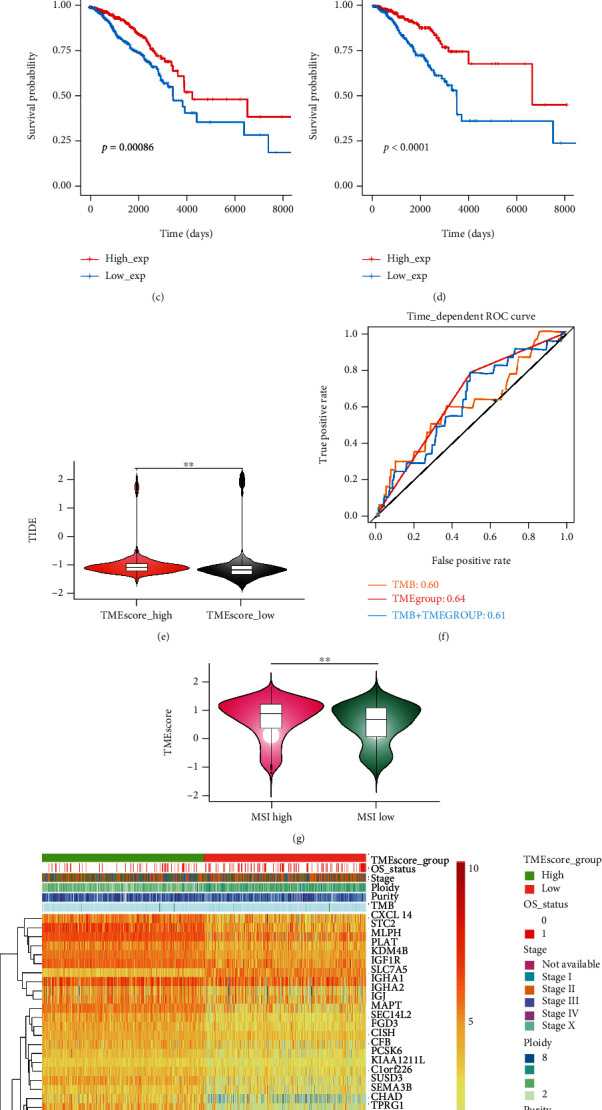
Comprehensive analysis results of tumor samples. (a) Volcano map of differentially methylated sites: 217 significantly different methylation sites were detected, and 67 methylation sites related to survival were obtained. (b) The survival analysis results of hsa-mic-1307 in different subgroups: the abscissa axis represents the survival time, and the ordinate axis represents the survival probability. The red curve represents high expression, and the blue curve represents low expression. (c) The survival analysis results of LRRC48 in different subgroups: the abscissa axis represents the survival time, and the ordinate axis represents the survival probability. The red curve represents high expression, and the blue curve represents low expression. (d) The survival analysis results of cg25726128 in different subgroups: the abscissa axis represents the survival time, and the ordinate axis represents the survival probability. The red curve represents high expression, and the blue curve represents low expression. (e) Immunotherapy efficacy score calculated by TMEscore group: the abscissa axis represents the TMEscore subgroup, and the ordinate axis represents TIDE. ∗∗ represents that the analysis result is statistically significant. (f) Using ROC analysis to evaluate the predictive ability of TMB, TMEgroup, and TMB+TME group on the effect of immunotherapy. (g) The relationship between MSI and TMEscore: the abscissa axis represents different MSI subgroups, and the ordinate axis represents TMEscore. ∗∗ represents that the analysis result is statistically significant. (h) Comprehensive genome landscape of BRCA (47 survival-related genes, *P* < 0.01).

In order to explore differences in the methylome of breast cancer samples, we downloaded TCGA-BRCA methylation chip data and analyzed 760 samples with significantly high or low TMEscores (adj.*P* < 0.05, absolute difference value > 0.15, Methods, Figure 6(a)). Identifying miRNAs, mRNAs, and methylation sites that were correlated with TMEscores allowed us to proceed to determine whether or not these factors were correlated with the survival. 217 significantly different methylation sites were detected.

#### 3.3.4. Survival Analysis

According to the expression values of the above differential miRNA, genes, methylation sites, or methylation levels (whether they were greater than the median of the expression value in all samples), the samples were divided into 2 groups. Log-rank test was used to determine whether these differential mRNA, miRNA, and methylation sites were related to the survival. A total of 6 miRNAs, 124 genes, and 67 methylation sites related to the survival (*P* < 0.05) were obtained. For example, we found that miRNA hsa-mir-1307, gene LRRC48, and methylation site cg25726128 were significantly correlated with the survival of BRCA patients (Figures 6(b)–6(d)).

### 3.4. The Correlation between TME and Immune Checkpoint Inhibitor (ICI) Treatment

Next, we used TIDE (tumor immune dysfunction and exclusion, http://tide.dfci.harvard.edu/) to evaluate the clinical effects of immune checkpoint inhibitor (ICI) therapy in the two sets of BRCA samples. As shown in (Figure 6(e)), it could be seen that the TIDE score of the high TMEscore group was significantly higher than that of the low TMEscore group (wilcox.test, *P* value = 1.067e-12).

ROC was used to evaluate the predictive ability of TMB, TME group, TMB+TME group on the effect of immunotherapy (TIDE score was used as the immune efficacy score). The result revealed that TME group was better than TMB as a prognostic tool (roc.test, *P* value = 0.003749, Figure 6(f)).

#### 3.4.1. The Relationship between TME and MSI

It was reported that patients with MSI-H had a better prognosis [39]. Therefore, combined with the TMEscore high samples with better prognosis in this analysis, the MSI was analyzed. According to the MSI score results predicted by TIDE, the samples were divided into MSI high and low groups (high: 146; low: 911). We found that the TMEscores responding to the two sets of MSI-high/low samples were significantly different (wilcox.test, *P* = 2.589e − 09) (Figure 6(g)).

#### 3.4.2. Comprehensive Genome Landscape of Tumor Samples

According to the TMEscore grouping information (TMEscore, TMEscore_group), mutations (purity, ploidy, and TMB), and clinical information (STAGE OS_STATUS) of the sample, the comprehensive genome landscape of the tumor sample was depicted, which is shown Figure 6(h).

## 4. Discussion

Breast cancer is a heterogeneous disease with highly variable clinical outcomes [40–42]. In the United States, although the incidence rate of breast cancer has slightly increased in recent years, the overall mortality rate has been in decline [43]. The overall decline in the mortality of breast cancer patients could be credited to committed and diverse studies in this field [6–8, 40–45]. Recently, the concept of comprehensive immunotherapy for breast cancer had been gradually developed [45, 46], and immune-related indicators have been demonstrated to have a significant impact on the prognosis of various tumors including breast cancer [19–22]. We therefore profiled the tumor immunological microenvironment and determined its predictive value for breast cancer patients.

It is reported that a large number of macrophage infiltration indicate a poor prognosis for cancer patients [47]. In vivo studies suggest that macrophages can stimulate angiogenesis, promote tumor cell extravasation, increase tumor cell migration, and further promote the occurrence and malignant progression [47]. Immunotherapy targeting macrophages may bring new hopes to cancer patients [48]. Therefore, clarifying the relationship between macrophages and the prognosis of cancer patients in clinical samples may have a significant role in promoting drug targeting macrophages. Through a comprehensive analysis of the infiltration status of 22 immune cells (S Figure 1A) and their correlation with survival (S Figure 1B), we found that resting Mast cell was a favorable factor for the OS, and M0 macrophage was a risk factor for the OS, which were rarely reported in breast cancer [40]. Further analysis also showed that the higher M0 macrophage infiltration was correlated to the worse prognosis of breast cancer patients (Figures 1(d) and 1(e)). Therefore, the M0 macrophage infiltration ratio may be used as an indicator to predict the prognosis of breast cancer patients in the future.

TMEscore, rarely reported in breast cancer, was considered to be related to the prognosis of cancer patients [10]. We therefore used a similar approach to evaluate the relationship between TMEscore and the survival (S Figure 2A) [10]. Our analysis revealed that 177 genes of interest were significantly enriched in immune-related pathways such as lymphocyte migration, lymphocyte chemotaxis, and leukocyte chemotaxis (S Figure 2B). The Cox regression model was used to determine the relationship between those genes and the survival of samples. All genes were divided into 2 categories according to the coefficient value of them, and all samples were scored by TMEscore using the TMEscore calculation formula. We found that there was a positive correlation between TMEscore and the survival of patients (S Figure 2C), which was never been reported by others.

Further validation of TMEscore was conducted in different subgroups (S Figure 3). All analysis results indicated that the obtained TMEscore might be a good characterization of the prognosis of clinical samples regarding their survival (S Figure 3). The evaluation effect of TMEscore model also indicated that TMEscore was a very good prognostic indicator for breast cancer patients (Figure 2(a)). Our strata analysis found that TMEscores were different in different pathological subgroups (luminal A, luminal B, basal, and Her 2; Figure 2(c)). The subgroup analysis based on TMEscore also suggested that TMEscore was a much better indicator for evaluating the prognosis and survival of breast cancer patients than their pathological phenotypes (Figures 2(d)–2(f)). Furthermore, there was a significantly negative correlation between TMEscore and TMB (Figure 2(b); *P* < 2.2 × 10^−16^). Although the relationship between TMEscore and the prognosis has been reported in other malignant tumors [10, 49], this is the first report in breast cancer. These findings lay the foundation for the clinical application of TMEscore in the future.

Through mutation analysis, we found that missense mutations were the most common alterations in BRCA [50, 51], and C>T was the most common type of SNP mutation in both high TMEscore and low subgroup (S Figure 4A and S Figure 4B). Similarities between the mutation characteristics of the TMEscore groups and those of the COSMIC mutation signature were analyzed and provided (S Figure 4C and S Figure 4D). Through analysis, we found that the mutation profile of the high TMEscore group was mainly related to Signatures 2, 10, 30, 24, and 1 (S Figure 4C), while the low TMEscore group was mainly related to Signatures 2, 3, and 6 (S Figure 4D). Among them, Signature 24 was firstly found and reported in breast cancer (https://cancer.sanger.ac.uk/signatures_v2/matrix.png) [52–54]. PIK3CA, reported in other studies [55, 56], was the gene with the highest mutation rate in the high TMEscore group (47%; Figure 3 B1), while TP53 was the most commonly mutated in the low TMEscore group (59%; Figure 3 B2) [57, 58]. PIK3CA, MUC4, TP53, KMT2C, and GATA3 were found to be significantly different between the high TMEscore and the low TMEscore group (Figure 3(c)). Among top 10 mutation genes, MUC4 was the gene with the highest mutation rate (24%; Figure 3 A6), which was reported to be uncorrelated with prognosis [59, 60].

Copy number alteration was a driving factor for the occurrence and development of cancer, and it played an important role in the entire developmental process of cancer [52, 61]. Consistent with earlier reports, the most prevalent somatic copy number alteration (SCNA) in our study was either very short (focal) or arm-level [61]. Through analysis, we found that 1q amplification was the most common arm-level SCNAs, regardless of TMEscore (Figure 4(a)). Incidentally, chromosome 1q21.3 amplification was considered to be a trackable biomarker and actionable target for breast cancer recurrence [62]. 16q deletion was the most common arm-level SCNA among all samples (Figure 4(a)), while 17p deletion is the most frequently occurring SCNA in the low TMEscore subgroup. Moreover, we found that in most cases, the incidence frequency of deletion or amplification in the same chromosome region seems to be significantly higher in the low TMEscore subgroup than that of the high TMEscore subgroup, which was rarely reported in other studies (Figure 4(a)) [52, 61–63]. Similar status could also be seen in focal SCNAs (Figures 4(b) and 4(c)). In other words, genome amplification or deletion alterations are much more likely to be found in the low TMEscore subgroup (Figures 4(a)–4(c)).

83 amplification and 36 deletion loci belonging to SCNA regions were found, and most of them have been reported in other studies [64]. However, the relationship between genes in these regions and the prognosis of breast cancer patients has not been systematically reported [61–64]. Pancancer survival analysis of genes across breast cancer in focal SCNAs was conducted and summarized (Tables 1 and 2 and S Figure 5). A total of 95 amplified genes and 169 deletion genes in the high TMEscore group were found to be significantly related to the prognosis of breast cancer (Table 1; S Figure 5), while 61 amplified genes and 174 deletion genes in the low TMEscore group were identified (Table 2; S Figure 5). In the high TMEscore group, the chromosomal loci with the highest frequency of amplified genes was located at 8q24.21 (*n* = 16; S Figure 5A1), and the highest frequency of deleted genes was located at 1p36.11 (*n* = 52; S Figure 5A2), while in the low TMEscore group, the chromosomal loci with the highest frequency of amplified genes were found at 10p14 (*n* = 11, S Figure 5B1), and the highest frequency of deleted genes was found at 1p36.13 and 3p14.2 (*n* = 36; S Figure 5B2). The above results in SCNAs regions were all discovered and reported for the first time. Our results would provide a framework for clinical work, especially for the comprehensive evaluation of the prognosis for breast cancer patients.

Through comprehensive analysis and evaluation, abnormal changes in tumor purity and ploidy, which were reported in other cancers [65–67], were also detected in breast cancer. This discovery suggested that genome disorder was a common phenomenon during carcinogenesis, but the correlation between them and breast cancer remains to be elucidated (Figures 4(d) and 4(e)).

We found that hsa-mir-19a-3p, hsa-mir-30a-3p, hsa-mir-9-5p, hsa-mir-105-3p, and hsa-mir-18a-5p were enriched both in cancer and immune pathways (Figure 5(a)). hsa-mir-30a-3p [68], hsa-mir-9-5p, and hsa-mir-18a-5p have ever been reported in breast cancer [69]. hsa-mir-19a-3p, hsa-mir-30a-3p, and hsa-mir-9-5p were validated to be significantly correlated with the prognosis of breast cancer (http://starbase.sysu.edu.cn/panMirSurvivalExp.php#). Through mRNA differential expression analysis, these corresponding genes were mainly upregulated in the high TMEscore group (Figures 5(b) and 5(c)). Among the top 9 upregulated genes, 6 of them (LRRC48, CFAP69, BTG2, KDM4B, TPRG1, and SCUBE2) were found to be relevant to the survival of breast cancer patients (Figure 6(c); http://starbase.sysu.edu.cn/panGeneSurvivalExp.php). Differentially expressed genes were enriched in the cell cycle, cell division, and other related pathways, indicating that major different biological processes between the two sets of samples (high/low TMEscore) lie in cell division and proliferation (Figures 5(d) and 5(e)). Methylation is a common epigenetic change that affects the development, prognosis, and treatment of tumors [70–72]. We found 217 significantly different methylation sites in our datasets (Figure 6(a)). Furthermore, 6 miRNAs, 124 genes, and 67 methylation sites related to survival were obtained. has-mir-1307 was significantly correlated to the survival (Figure 6(b)) [73]. From Figure 6(d), it could be found that cg25726128 was significantly correlated with the survival of BRCA patients. Furthermore, LRRC48, CFAP69, and cg25726128 were first discovered and reported to be related to the survival of breast cancer patients (Figures 6(c) and 6(d)). These new findings would be helpful for us to conduct more in-depth research on the mechanism of breast cancer in the future.

As shown in (Figure 6(e)), it could be seen that the TIDEscore of the high TMEscore group was significantly higher than that of the low TMEscore group (*P* value = 1.067e-12). The higher tumor TIDE prediction score was related to the poorer response to immune checkpoint inhibitor therapy. In the previous analysis, the high TMEscore group has a better prognosis, and the low TMEscore group has a poor prognosis. Corresponding to the results of this TIDE assessment, it showed that the high TMEscore group had a good prognosis, but the low TMEscore group had a better response on immune checkpoint inhibitor (ICI) therapy. ROC was used to evaluate the predictive ability of TMB [38], TME group, and TMB+TME group on the effect of immunotherapy (Figure 6(f)). We found that TME was better than TMB as a prognostic tool (roc.test, *P* value = 0.003749). TMEscores responding to the two sets of MSI were significantly different (wilcox.test, *P* = 2.589e − 09) (Figure 6(g)). Negative correlation between TMEscore and TMB is displayed in Figure 2(b) (*P* < 2.2 × 10^−16^). Although significant correlation between TMB and MSI was reported in colorectal and pancreatic cancer [74], the relationship between TMEscore and MSI in BRCA still needed to be validated. But this would not weaken the potential of TMEscore as an indicator for the prognostic evaluation in breast cancer patients (Figure 6(f)). It could be found that genomic alterations in those two TMEscore groups were significantly different from each other (Figure 6(h)). In a word, it showed that the TME method could be effectively used to predict tumor prognosis and the responsiveness to ICI therapy.

Compared with traditional scoring methods, the advantage of the TMEscore scoring method is that more cells are included in the score [10, 38]. Twenty-two kinds of immune cells are included in this project [10]. We first clustered them according to the immune infiltration situation and then screened the genes related to infiltration. In this step, the candidate gene set was expanded to ensure the accuracy. Finally, the gene expression grade index (GGI) score was used to effectively simplify the model and facilitate calculation [37]. Compared with GGI, TMEscore has fewer requirements for the number of samples, and the calculation process is much simpler.

## 5. Conclusion

TMEscore could be effectively used to predict tumor prognosis and the efficacy of ICI. Signature 24 was first found in breast cancer. In focal SCNAs, a total of 95 amplified genes and 169 deletion genes in the high TMEscore group are found to be significantly related to the prognosis of breast cancer patients, while 61 amplified genes and 174 deletion genes in the low TMEscore group were found. LRRC48, CFAP69, and cg25726128 were first discovered and reported to be related to the survival of breast cancer patients.

## Figures and Tables

**Table 1:** The distribution of genes that are significantly related to the survival of breast cancer patients in different chromosomes (high TMEscore group).

Type	Cytoband	*q* value	Residual *q* value	Wide peak boundaries	Genes in wide peak (pancancer survival analysis of genes, log-rank *P* < 0.05)
Amplification	11q13.2	5.19E-30	1.26E-14	chr11:67149025-67239409	CARNS1, TBC1D10C
	8q24.21	3.57E-14	1.77E-10	chr8:112209072-132893892	NDUFB9, TAF2, EIF3H, ZHX1, MRPL13, GSDMC, DSCC1, DERL1, UTP23, C8orf76, NSMCE2, LINC00536, MAL2, TMEM65, FAM84B, and FAM91A1
	1q32.1	4.94E-13	1.37E-07	chr1:200049409-207682721	C4BPA, CACNA1S, MYBPH, PIGR, BTG2, IL24, UBE2T, LGR6, and SYT2
	12q15	6.45E-05	6.45E-05	chr12:68571667-71730803	CCT2, IL26
	19q13.43	0.0004018	0.0004018	chr19:57711362-58759151	ZNF586, ZNF552, ZNF671, ZNF587, ZSCAN1, and ZNF773
	8q21.13	2.72E-09	0.0013026	chr8:80930566-81905402	TPD52
	15q26.3	0.0015596	0.0015596	chr15:97773672-99710785	IGF1R
	1q42.3	1.62E-08	0.0045109	chr1:224184822-249250621	KCNK1, GPR137B, CDC42BPA, DEGS1, KMO, EXO1, PGBD5, and LINC00184
	11q13.4	3.32E-15	0.010598	chr11:75104213-75489974	KLHL35
	11q12.2	0.0050379	0.025986	chr11:60965301-61392747	CPSF7
	20q13.31	7.78E-09	0.10561	chr20:55905630-56139966	MTRNR2L3
	20q13.32	5.47E-08	0.12447	chr20:57697096-58759307	SYCP2, ZNF831, and C20orf197
	6p24.1	0.13739	0.13739	chr6:8334749-26341802	HIST1H1T, MAK, CD83, ELOVL2, MRS2, and MBOAT1
	20q13.33	1.50E-06	0.16137	chr20:60450310-63025520	OPRL1, PTK6, OSBPL2, DIDO1, RTEL1, and SLC17A9
	16p13.3	0.21045	0.21045	chr16:1-11927917	hsa-mir-940, ABAT, OR1F1, SRL, HBM, IGFALS, SOCS1, RHBDL1, RAB26, SNORD60, TBC1D24, ROGDI, PRSS27, C16orf89, and BCAR4
	1q21.3	3.75E-05	0.21456	chr1:154496590-155333859	RUSC1-AS1
	17q25.1	4.00E-09	0.22085	chr17:70557347-78812525	ITGB4, LGALS3BP, RPL38, DNAH17, SOCS3, ST6GALNAC2, CD300C, CCDC40, MYO15B, CBX2, KIF19, ENDOV, RAB37, C17orf99, and MIR4730

Deletion	11q23.1	6.20E-28	6.20E-28	chr11:103701096-127804389	APOA1, ARCN1, FXYD2, CD3D, CD3E, CD3G, CRYAB, HSPA8, RDX, TECTA, UPK2, UBE4A, VSIG2, BACE1, FXYD6, ARHGAP20, C11orf1, CLMP, APOA5, TTC36, OR6T1, CCDC153, OR10G7, DDI1, and OR8D4
	17p12	4.48E-14	4.48E-14	chr17:11939511-12020487	hsa-mir-744-3p
	8p21.2	3.38E-24	1.13E-10	chr8:19767860-28832196	CLU, EGR3, SLC18A1, TRIM35, BIN3, NUDT18, and C8orf58
	16q24.2	5.23E-06	5.23E-06	chr16:73537103-90354753	CYBA, RPL13, SLC7A5, CLEC3A, JPH3, VAT1L, DNAAF1, DYNLRB2, ADAMTS18, and FAM92B
	6q21	2.84E-09	2.79E-05	chr6:85623676-114360570	HDAC2, AMD1, NT5E, POU3F2, SNX3, RNGTT, FIG4, SEC63, PDSS2, MICAL1, RTN4IP1, and SCML4
	1p36.11	0.0001786	0.0001786	chr1:1-29548541	CA6, CASP9, RUNX3, CD52, CLCNKB, CNR2, CORT, NPPA, PAX7, PIK3CD, PTAFR, RPL11, TP73, RPL22, RPS6KA1, TNFRSF25, TNFRSF14, TNFRSF18, H6PD, SRRM1, SYF2, CHD5, SMPDL3B, CELA2B, ZNF593, ERRFI1, PQLC2, TRNAU1AP, XKR8, PNRC2, KIF17, NBPF1, MIIP, LIN28A, LINC00115, MORN1, GPR157, ACTL8, EFHD2, TAS1R3, PLEKHN1, UBXN11, C1orf158, FHAD1, RBP7, ACTRT2, GPR153, PLA2G2C, RNF207, SNORA59B, TTC34, and MIR4684
	13q14.2	0.0013005	0.0013005	chr13:48870266-51811240	PHF11, CTAGE10P, and ARL11
	3p21.31	0.0015062	0.001506	chr3:41907969-75074934	CCR3, CCR5, FHIT, FLNB, GPR27, ITIH1, CISH, ITIH3, MST1, PDHB, RPL29, TDGF1, UBA7, USP4, SEMA3B, HYAL3, HYAL2, PARP3, RBM6, RBM5, CCR9, TMEM115, MIR4443, TUSC2, EIF4E3, C3orf62, SPINK8, CDHR4, ABHD14A, PTPN23, RBM15B, ARHGEF3, SS18L2, SHISA5, ZMYND10, HEMK1, IP6K2, PHF7, P4HTM, SNRK, ANO10, SEMA3G, KIF9, CAMKV, LRRC2, FAM3D, and SNTN
	6q27	1.76E-07	0.0036694	chr6:165745183-171115067	CCR6, DACT2, and C6orf120
	11q13.1	0.11052	0.11052	chr11:64565960-65674152	MIR4690, CTSW
	12p13.1	0.21108	0.21108	chr12:10747241-18912988	ARHGDIB, PRB1, TAS2R7, GPRC5D, BCL2L14, TAS2R43, and TAS2R30

**Table 2:** The distribution of genes that are significantly related to the survival of breast cancer patients in different chromosomes (low TMEscore group).

	Cytoband	*q* value	Residual *q* value	Wide peak boundaries	Genes in wide peak (pancancer survival analysis of genes, log-rank *P* < 0.05)
Amplification	8q23.3	3.95E-64	1.42E-13	chr8:116404190-117369718	LINC00536
	1q42.3	1.69E-11	6.15E-10	chr1:233225485-249250621	KCNK1; GPR137B; EXO1; and LINC00184
	12q15	2.97E-08	2.97E-08	chr12:69232907-70028065	CCT2
	1q23.3	1.38E-16	4.61E-07	chr1:160766283-161196692	TOMM40L
	19q12	6.74E-07	6.74E-07	chr19:30071032-30519016	CCNE1
	17q23.3	2.32E-34	3.72E-06	chr17:61938003-62464820	GH2
	8p11.21	4.68E-13	8.02E-06	chr8:41464872-42334787	PLAT;SLC20A2
	15q26.3	1.46E-05	1.46E-05	chr15:98898260-99719772	IGF1R
	10p14	0.00010116	0.00010116	chr10:2133333-14235192	CALML3; PRKCQ; AKR1C3; CDC123; PITRM1; NUDT5; SEPHS1; MCM10; DHTKD1; SFMBT2; and AKR1E2
	6p23	0.0015466	0.0015466	chr6:8294791-22998441	MAK; CD83; ELOVL2; MBOAT1; and HDGFL1
	20q13.32	1.56E-13	0.0034782	chr20:55360152-57763459	hsa-mir-4325; MTRNR2L3
	8q21.13	8.30E-23	0.0041212	chr8:80799143-81914705	TPD52
	17q25.3	2.00E-18	0.0043376	chr17:77706199-77827008	CBX2
	10q22.3	0.021087	0.021087	chr10:79530228-82012440	RPS24; SFTPD; and EIF5AL1
	3q26.32	2.40E-07	0.021413	chr3:176689515-179942551	ZMAT3
	20q13.33	2.33E-10	0.029112	chr20:62160482-63025520	OPRL1; PTK6; and RTEL1
	5p15.33	0.030952	0.030952	chr5:1-4423291	SLC6A3; AHRR; SLC6A19; and SDHAP3
	8q21.3	8.78E-31	0.035914	chr8:90681176-94209709	DECR1; OTUD6B
	4q13.3	0.11535	0.11535	chr4:73718359-75035796	CXCL1; CXCL2; CXCL3; and COX18
	13q34	0.12313	0.12313	chr13:97958870-115169878	GPR18; ZIC2; IRS2; FARP1; KDELC1; CARS2; DAOA; LINC00346; UBAC2-AS1; and MIR548AN
	3q26.2	1.34E-06	0.14005	chr3:161762173-175078479	CLDN11; WDR49
				
	8p21.3	2.12E-52	8.34E-20	chr8:21912133-25692095	EGR3; BIN3; NUDT18; and C8orf58
	1p36.13	2.15E-12	2.15E-12	chr1:1-24656091	CA6; CASP9; CLCNKB; CNR2; CORT; NPPA; PAX7; PIK3CD; RPL11; RPL22; TNFRSF25; TNFRSF14; TNFRSF18; H6PD; CHD5; CELA2B; ERRFI1; PNRC2; NBPF1; KIF17; MIIP; EFHD2; LINC00115; MORN1; GPR157; ACTL8; TAS1R3; PLEKHN1; C1orf158; RBP7; ACTRT2; RNF207; PLA2G2C; SNORA59B; TTC34; and MIR4684
	3p14.2	2.73E-11	2.73E-11	chr3:48386251-69373975	CISH; FHIT; FLNB; ITIH1; ITIH3; MST1; PDHB; RPL29; UBA7; USP4; IFRD2; SEMA3B; HYAL3; HYAL2; PARP3; RBM6; RBM5; TMEM115; TUSC2; ABHD14A; RBM15B; ARHGEF3; SHISA5; ZMYND10; HEMK1; IP6K2; PHF7; P4HTM; SEMA3G; CAMKV; FAM3D; SNTN; C3orf62; CDHR; SNORD69; and ESRG
	19p13.3	8.22E-10	8.22E-10	chr19:1-1942022	AZU1; CIRBP; GZMM; APC2; CIRBP-AS1; and ODF3L2

Deletion	5q13.3	3.75E-05	3.75E-05	chr5:53747308-89283337	BTF3; CKMT2; GZMA; GZMK; TAF9; ENC1; HSPB3; EDIL3; NSA2; ANKRD55; NBPF22P; and LINC00461
	6q26	4.72E-05	4.72E-05	chr6:156683000-171115067	CCR6; IGF2R; TCP1; DYNLT1; ARID1B; SERAC1; DACT2; OSTCP1; TMEM242; and C6orf99
	11q23.2	1.08E-14	5.22E-05	chr11:101000626-118347999	BIRC3; APOA1; FXYD2; CD3D; CD3E; CD3G; CRYAB; RDX; MMP20; UBE4A; BACE1; FXYD6; ARHGAP20; C11orf1; APOA5; DDI1; and FXYD6-FXYD2
	11q25	1.87E-14	0.00011266	chr11:132748397-135006516	GLB1L3
	11p15.5	0.00022487	0.00022487	chr11:1-549958	IFITM1; SCGB1C1; and NLRP6
	15q11.2	0.0003078	0.0003078	chr15:1-43514475	LTK; RAD51; RASGRP1; CHP; RPAP1; PPP1R14D; PAK6; NIPA2; TUBGCP5; NIPA1; FSIP1; C15orf53; GOLGA8B; MIR626; SNORD115-16; SNORD115-33; and TMCO5B
	2q37.3	0.0049716	0.0049716	chr2:234295756-243199373	HDLBP; PDCD1; RAMP1; PASK; SH3BP4; ANKMY1; MLPH; and MSL3P1
	10q26.3	0.00033563	0.013065	chr10:134143498-135534747	INPP5A; UTF1; NKX6-2; PRAP1; and DUX4L2
	18q23	0.039797	0.039797	chr18:62564783-78077248	CD226
	16q24.3	0.058632	0.058632	chr16:76942801-90354753	CYBA; RPL13; SLC7A5; CLEC3A; VAT1L; DYNLRB2; DNAAF1; and FAM92B
	4p16.3	0.13214	0.13214	chr4:1-37273894	DHX15; MYL5; FGFBP1; FAM184B; DCAF16; CCDC96; DOK7; and DTHD1
	20p13	0.20564	0.20564	chr20:1-637696	ZCCHC3; DEFB132

## Data Availability

All data involved in this study can be downloaded from the websites mentioned in the above or obtained free of charge by contacting the corresponding author.

## Abbreviations

TME: Tumor microenvironment
iTME: Immune tumor microenvironment
LM22: Leukocyte signature matrix 22
DEG: Differentially expressed genes
UCSC: University of California, Santa Cruz
TCGA: The Cancer Genome Atlas
GEO: Gene Expression Omnibus
CNV: Copy number variation
GDC: Genomic Data Commons
CDF: Cumulative distribution function
SNP: Single nucleotide polymorphisms
GISTIC: Genomic Identification of Significant Targets in Cancer
TIDE: Tumor immune dysfunction and exclusion
TMB: Tumor mutation burden
MCRs: Minimum common regions
SCNAs: Somatic copy number alterations
ICI: Immune checkpoint inhibitor
GGI: Gene expression grade index.

## Additional Points

*Confirmation*. We confirmed that the institution was aware of the work being submitted to the Journal. All the analyses were performed in Jinan central hospital affiliated to Shandong University and Shandong First Medical University, which were completed in Nov 28, 2021. The corresponding authors employments were listed in right affiliated institution. The contact details of the corresponding authors had been provided.

## Supplementary Materials

Supplementary Materials Supplementary Table 1: the basic characteristics of the 4 datasets: TCGA-BRCA, GSE96058, GSE124647, and GSE25066. Supplementary Figure 1: infiltrating cells in the TME. (A) The distribution ratio of 22 kinds of immune cells (B cells memory, dendritic cells activated, macrophages M0, etc.) in different samples: the ordinate represents the proportion of different immune cells, the abscissa represents different samples, and the color represents the type of immune cells. (B) The relationship between 22 kinds of immune cells and their relationship with survival (the larger the dot indicates the more related to survival, the thickness of the line indicates the strength of cell correlation). Supplementary Figure 2: the relationship between TMEscore and survival. (A) Consistent clustering results of differential genes: ConsensusClusterPlus was used for unsupervised class discovery (1000 iterations, *k* = 1 : 10). The optimal *k* value of 3 was determined using the elbow method and gap statics, combined with the correlation between the final classification and survival. The limma package of R was used to screen different types of differentially expressed genes (*P* < 0.05, |log2FC| > log2 (1.5)). 522 differentially expressed genes were obtained, and unsupervised cluster analysis was performed, and the samples were divided into three categories. (B) Functional enrichment analysis results of signature genes: functional enrichment analysis on 177 nonredundant genes using R ClusterProfiler revealed that this gene set was significantly enriched in immune-related pathways such as lymphocyte migration, lymphocyte chemotaxis, and leukocyte chemotaxis. (C) Survival analysis results of all enrolled samples based on TMEscore: a Cox regression model was used to determine the relationship between DEGs and the survival of samples. Next, genes were divided into 2 categories according to their coefficient values, and samples were divided into two groups based on high or low calculated TMEscores. Supplementary Figure 3: validation and model evaluation using TCGA-BRCA, GSE124647, and GSE25066. (A) Survival analysis for TCGA-BRCA based on TMEscore model: the red curve represents the high TMEscore group, and the blue curve represents the low TMEscore group; the ordinate axis represents the probability of survival, and the abscissa axis represents the survival days. (B) Survival analysis for GSE124647 (metastatic breast cancer) based on the TMEscore model: the red curve represents the high TMEscore group, and the blue curve represents the low TMEscore group; the ordinate axis represents the probability of survival, and the abscissa axis represents the survival days. (C1) Survival analysis for GSE25066 (chemotherapy) based on the TMEscore model: the yellow curve represents the high TMEscore group, and the blue curve represents the low TMEscore group; the ordinate axis represents the probability of survival, and the abscissa axis represents the survival days. (C2) ROC curve of GSE25066 (AUC = 0.612): the ordinate axis represents the detection sensitivity of the ROC curve, and the abscissa axis represents the detection specificity of the ROC curve. (D1) Survival analysis for GSE124647 (endocrine therapy) based on the TMEscore model: the yellow curve represents the high TMEscore group, and the blue curve represents the low TMEscore group; the ordinate axis represents the probability of survival, and the abscissa axis represents the survival days. (D2) ROC curve of GSE124647 (AUC = 0.557): the ordinate axis represents the detection sensitivity of the ROC curve, and the abscissa axis represents the detection specificity of the ROC curve. Supplementary Figure 4: the results of mutation spectrum analysis: nonnegative matrix decomposition of the frequency matrix with 1,042 samples in rows and 96 mutation types in the columns were performed, mutational characteristics of 3 somatic point mutations were extracted, and then, the similarity between the extracted features and the signature collected by cosmic was analyzed. (A) The frequency distribution of 96 mutation types in high TMEscore group: the most frequent type of mutation is C>T. (B) The frequency distribution of 96 mutation types in low TMEscore group: the most frequent type of mutation is C>T. (C) Similarity between the mutation characteristics of the high TMEscore group and those of the cosmic mutation signature: the mutation spectrum was mainly related to Signature 2, Signature 10, and Signature 30. (D) Similarity between the mutation characteristics of the low TMEscore group and those of the cosmic mutation signature: the mutation spectrum was mainly related to Signature 2, Signature 3, and Signature 6. Supplementary Figure 5: pancancer survival analysis of genes across cancers in SCNAs regions. The abscissa axis represents the chromosome locus, and the ordinate axis represents the number of genes locating in the corresponding chromosome locus. (A1) In the high TMEscore group, the distribution and number of amplified genes that are significantly related to the survival of breast cancer patients in different chromosomes. (A2) In the high TMEscore group, the distribution and number of deletion genes that are significantly related to the survival of breast cancer patients in different chromosomes. (B1) In the low TMEscore group, the distribution and number of amplified genes that are significantly related to the survival of breast cancer patients in different chromosomes. (B2) In the low TMEscore group, the distribution and number of deletion genes that are significantly related to the survival of breast cancer patients in different chromosomes.
