# Supplementary figures and images for: The Significance of Tumor Microenvironment Score for Breast Cancer Patients
Source: Biomed Res Int. 2022 Apr 28;2022:5673810. doi: 10.1155/2022/5673810 (PMC9071896; doi:10.1155/2022/5673810)

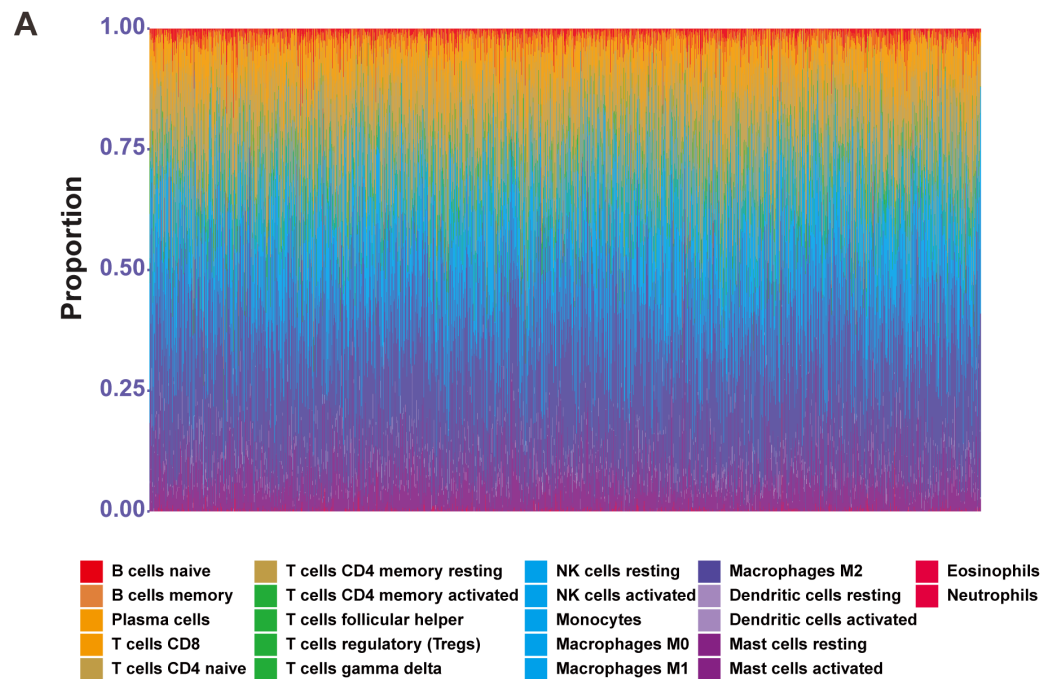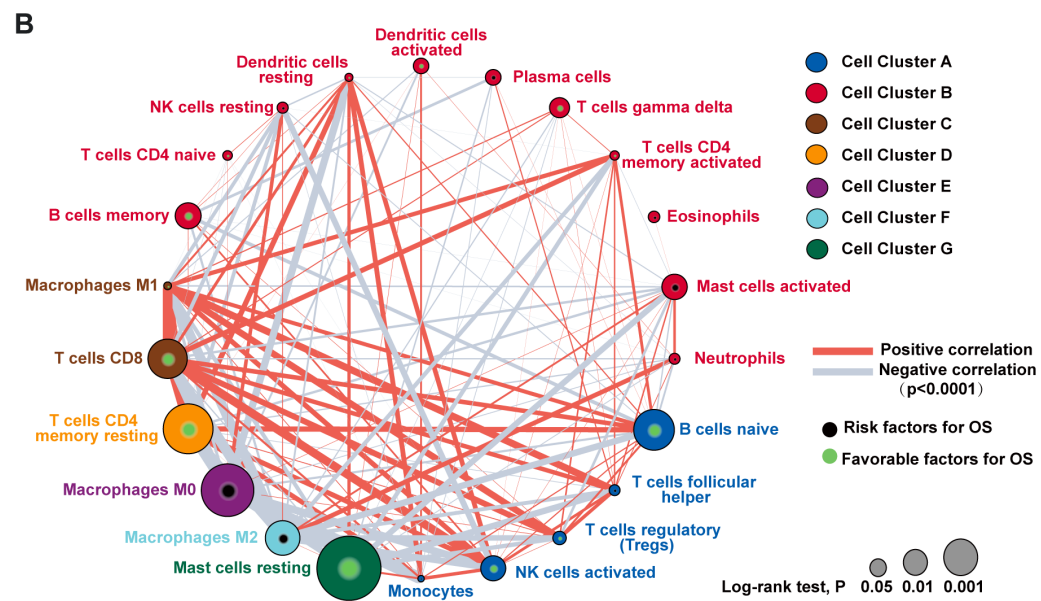

Supplement: Supplementary Materials — Supplementary Table 1: the basic characteristics of the 4 datasets: TCGA-BRCA, GSE96058, GSE124647, and GSE25066. Supplementary Figure 1: infiltrating cells in the TME. (A) The distribution ratio of 22 kinds of immune cells (B cells memory, dendritic cells activated, macrophages M0, etc.) in different samples: the ordinate represents the proportion of different immune cells, the abscissa represents different samples, and the color represents the type of immune cells. (B) The relationship between 22 kinds of immune cells and their relationship with survival (the larger the dot indicates the more related to survival, the thickness of the line indicates the strength of cell correlation). Supplementary Figure 2: the relationship between TMEscore and survival. (A) Consistent clustering results of differential genes: ConsensusClusterPlus was used for unsupervised class discovery (1000 iterations, k = 1 : 10). The optimal k value of 3 was determined using the elbow method and gap statics, combined with the correlation between the final classification and survival. The limma package of R was used to screen different types of differentially expressed genes (P < 0.05, |log2FC| > log2 (1.5)). 522 differentially expressed genes were obtained, and unsupervised cluster analysis was performed, and the samples were divided into three categories. (B) Functional enrichment analysis results of signature genes: functional enrichment analysis on 177 nonredundant genes using R ClusterProfiler revealed that this gene set was significantly enriched in immune-related pathways such as lymphocyte migration, lymphocyte chemotaxis, and leukocyte chemotaxis. (C) Survival analysis results of all enrolled samples based on TMEscore: a Cox regression model was used to determine the relationship between DEGs and the survival of samples. Next, genes were divided into 2 categories according to their coefficient values, and samples were divided into two groups based on high or low calculated TMEscores. [file 5673810.f1.zip › supplementary figures/Supplementary Figure 1.pdf]

**A**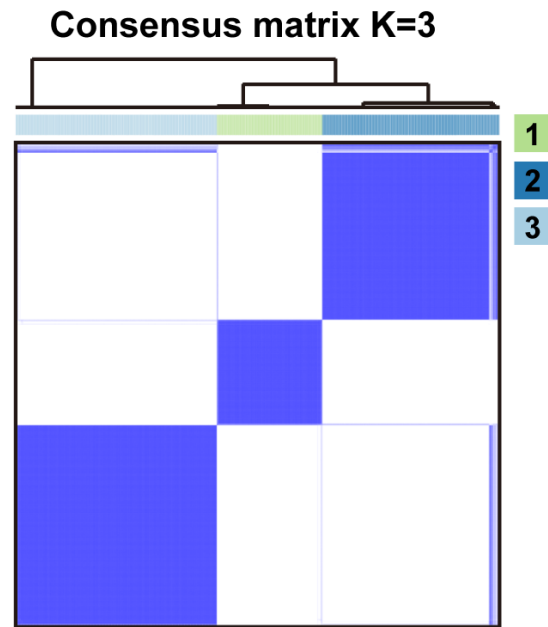**C**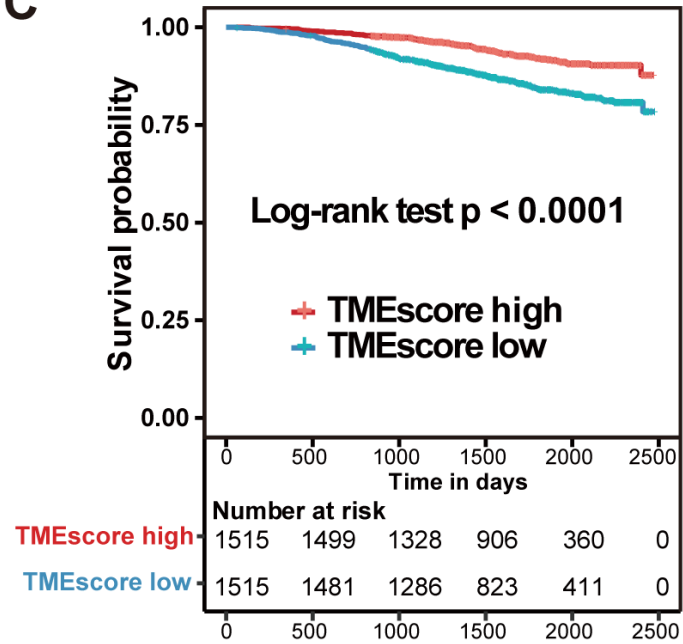**B**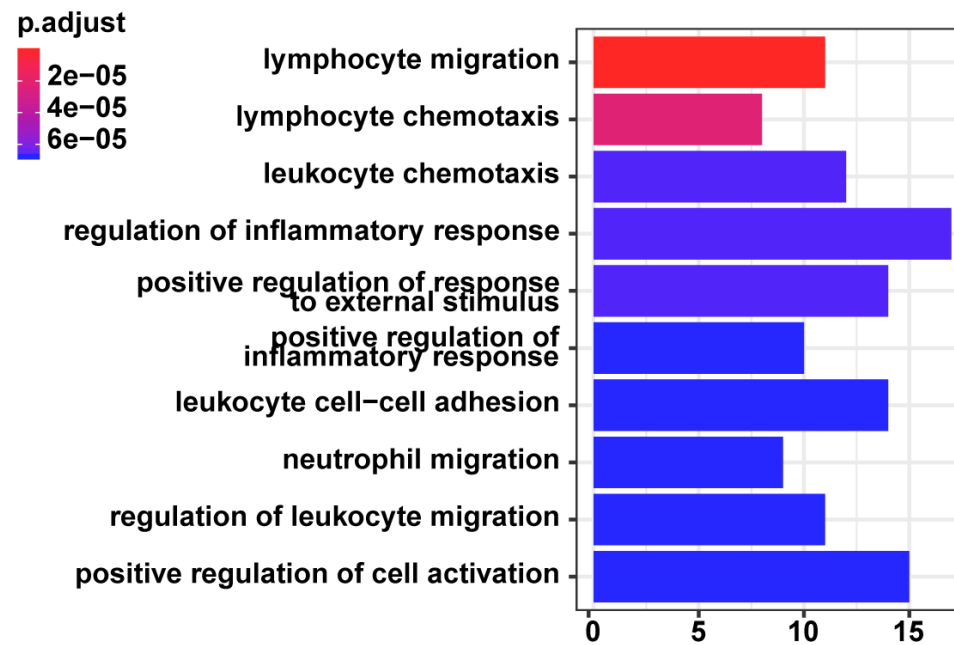

Supplement: Supplementary Materials — Supplementary Table 1: the basic characteristics of the 4 datasets: TCGA-BRCA, GSE96058, GSE124647, and GSE25066. Supplementary Figure 1: infiltrating cells in the TME. (A) The distribution ratio of 22 kinds of immune cells (B cells memory, dendritic cells activated, macrophages M0, etc.) in different samples: the ordinate represents the proportion of different immune cells, the abscissa represents different samples, and the color represents the type of immune cells. (B) The relationship between 22 kinds of immune cells and their relationship with survival (the larger the dot indicates the more related to survival, the thickness of the line indicates the strength of cell correlation). Supplementary Figure 2: the relationship between TMEscore and survival. (A) Consistent clustering results of differential genes: ConsensusClusterPlus was used for unsupervised class discovery (1000 iterations, k = 1 : 10). The optimal k value of 3 was determined using the elbow method and gap statics, combined with the correlation between the final classification and survival. The limma package of R was used to screen different types of differentially expressed genes (P < 0.05, |log2FC| > log2 (1.5)). 522 differentially expressed genes were obtained, and unsupervised cluster analysis was performed, and the samples were divided into three categories. (B) Functional enrichment analysis results of signature genes: functional enrichment analysis on 177 nonredundant genes using R ClusterProfiler revealed that this gene set was significantly enriched in immune-related pathways such as lymphocyte migration, lymphocyte chemotaxis, and leukocyte chemotaxis. (C) Survival analysis results of all enrolled samples based on TMEscore: a Cox regression model was used to determine the relationship between DEGs and the survival of samples. Next, genes were divided into 2 categories according to their coefficient values, and samples were divided into two groups based on high or low calculated TMEscores. [file 5673810.f1.zip › supplementary figures/Supplementary Figure 2.pdf]

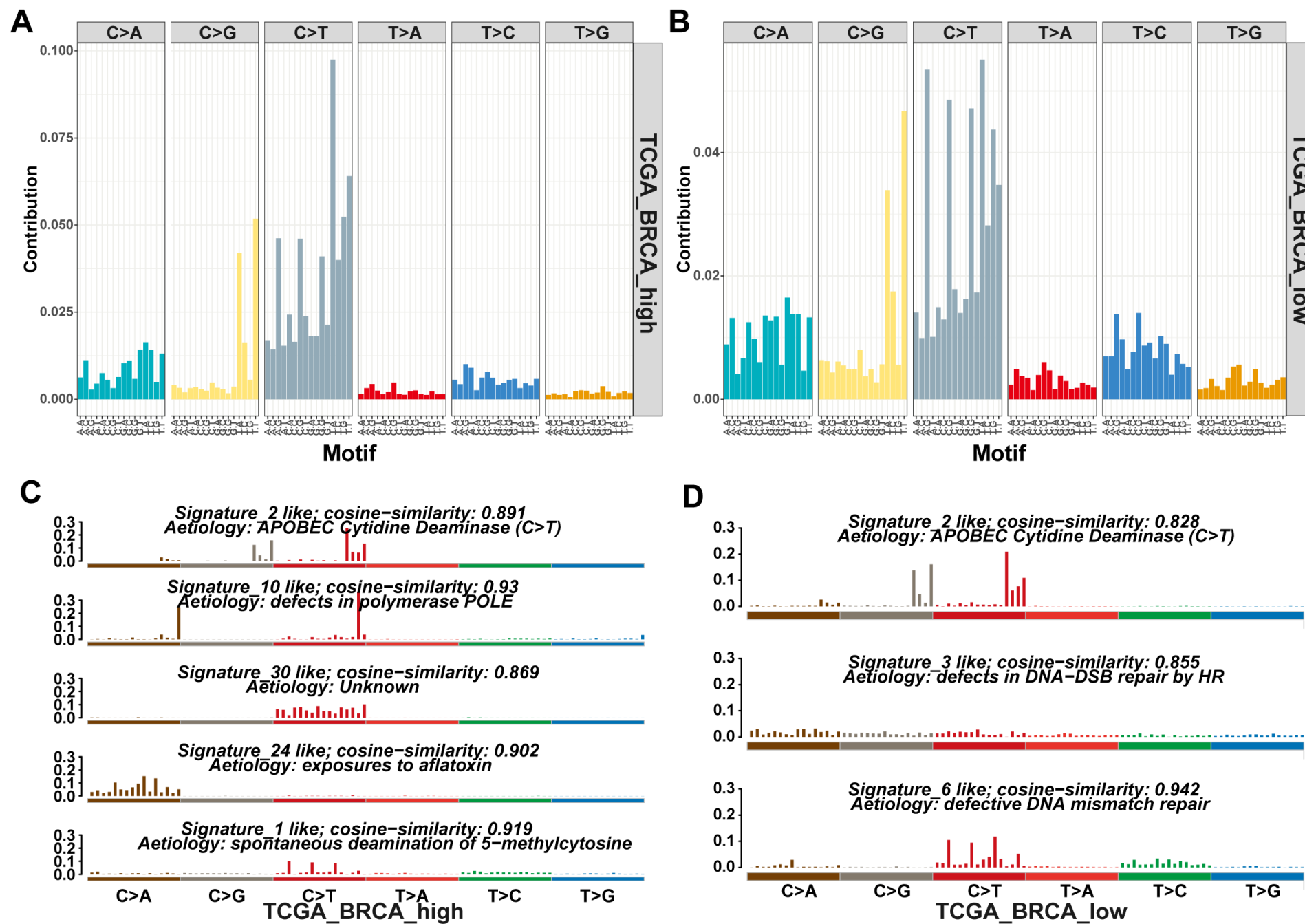

Supplement: Supplementary Materials — Supplementary Table 1: the basic characteristics of the 4 datasets: TCGA-BRCA, GSE96058, GSE124647, and GSE25066. Supplementary Figure 1: infiltrating cells in the TME. (A) The distribution ratio of 22 kinds of immune cells (B cells memory, dendritic cells activated, macrophages M0, etc.) in different samples: the ordinate represents the proportion of different immune cells, the abscissa represents different samples, and the color represents the type of immune cells. (B) The relationship between 22 kinds of immune cells and their relationship with survival (the larger the dot indicates the more related to survival, the thickness of the line indicates the strength of cell correlation). Supplementary Figure 2: the relationship between TMEscore and survival. (A) Consistent clustering results of differential genes: ConsensusClusterPlus was used for unsupervised class discovery (1000 iterations, k = 1 : 10). The optimal k value of 3 was determined using the elbow method and gap statics, combined with the correlation between the final classification and survival. The limma package of R was used to screen different types of differentially expressed genes (P < 0.05, |log2FC| > log2 (1.5)). 522 differentially expressed genes were obtained, and unsupervised cluster analysis was performed, and the samples were divided into three categories. (B) Functional enrichment analysis results of signature genes: functional enrichment analysis on 177 nonredundant genes using R ClusterProfiler revealed that this gene set was significantly enriched in immune-related pathways such as lymphocyte migration, lymphocyte chemotaxis, and leukocyte chemotaxis. (C) Survival analysis results of all enrolled samples based on TMEscore: a Cox regression model was used to determine the relationship between DEGs and the survival of samples. Next, genes were divided into 2 categories according to their coefficient values, and samples were divided into two groups based on high or low calculated TMEscores. [file 5673810.f1.zip › supplementary figures/Supplementary Figure 4.pdf]

**A**

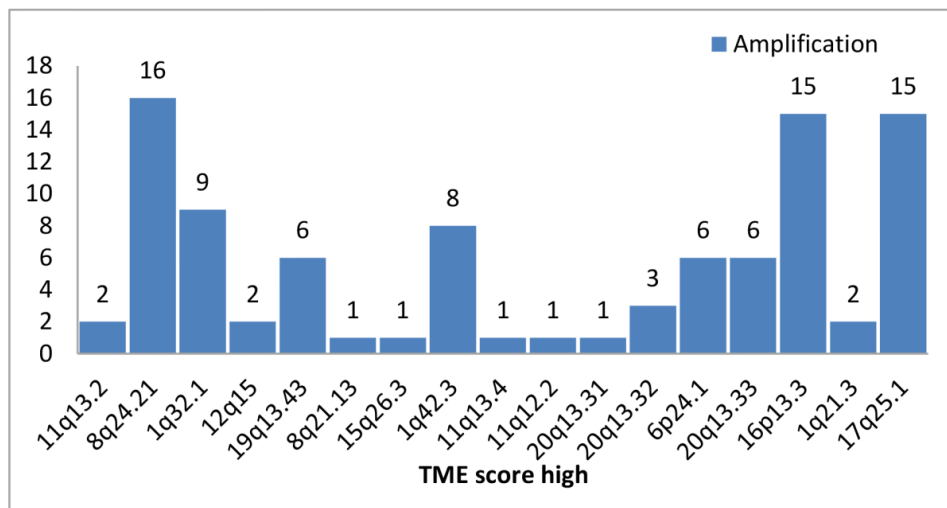

(A1)

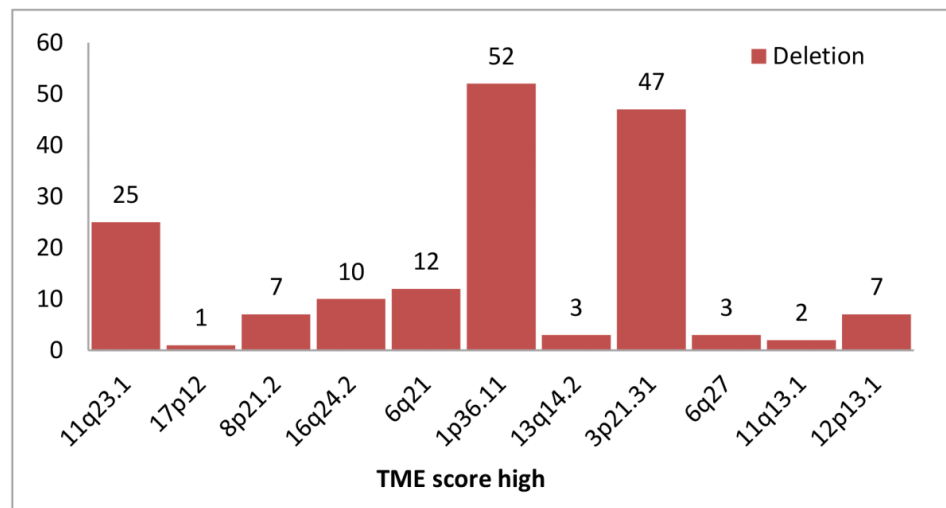

(A2)

**B**

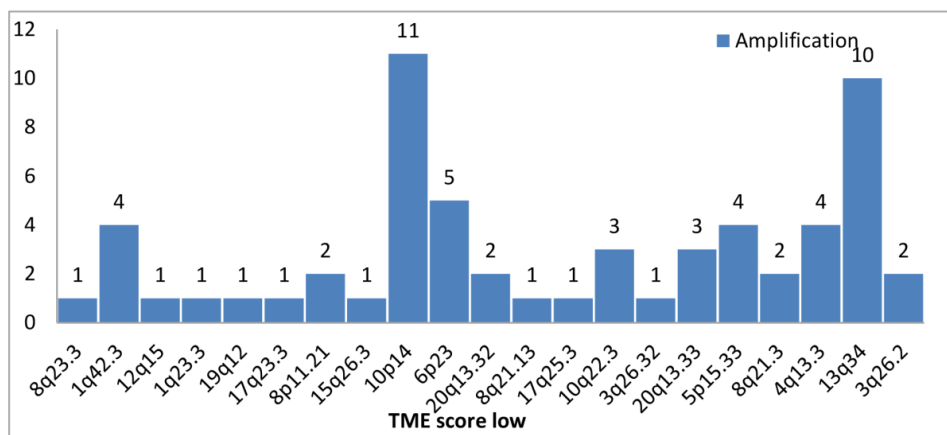

(B1)

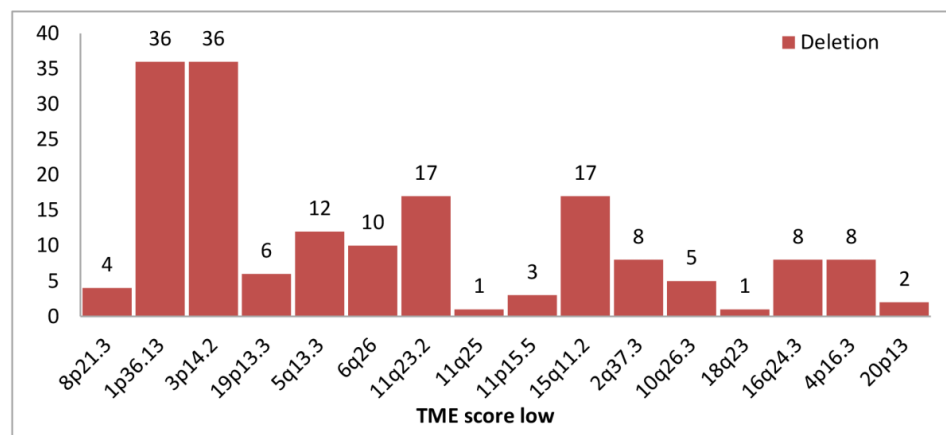

(B2)

Supplement: Supplementary Materials — Supplementary Table 1: the basic characteristics of the 4 datasets: TCGA-BRCA, GSE96058, GSE124647, and GSE25066. Supplementary Figure 1: infiltrating cells in the TME. (A) The distribution ratio of 22 kinds of immune cells (B cells memory, dendritic cells activated, macrophages M0, etc.) in different samples: the ordinate represents the proportion of different immune cells, the abscissa represents different samples, and the color represents the type of immune cells. (B) The relationship between 22 kinds of immune cells and their relationship with survival (the larger the dot indicates the more related to survival, the thickness of the line indicates the strength of cell correlation). Supplementary Figure 2: the relationship between TMEscore and survival. (A) Consistent clustering results of differential genes: ConsensusClusterPlus was used for unsupervised class discovery (1000 iterations, k = 1 : 10). The optimal k value of 3 was determined using the elbow method and gap statics, combined with the correlation between the final classification and survival. The limma package of R was used to screen different types of differentially expressed genes (P < 0.05, |log2FC| > log2 (1.5)). 522 differentially expressed genes were obtained, and unsupervised cluster analysis was performed, and the samples were divided into three categories. (B) Functional enrichment analysis results of signature genes: functional enrichment analysis on 177 nonredundant genes using R ClusterProfiler revealed that this gene set was significantly enriched in immune-related pathways such as lymphocyte migration, lymphocyte chemotaxis, and leukocyte chemotaxis. (C) Survival analysis results of all enrolled samples based on TMEscore: a Cox regression model was used to determine the relationship between DEGs and the survival of samples. Next, genes were divided into 2 categories according to their coefficient values, and samples were divided into two groups based on high or low calculated TMEscores. [file 5673810.f1.zip › supplementary figures/Supplementary Figure 5.pdf]

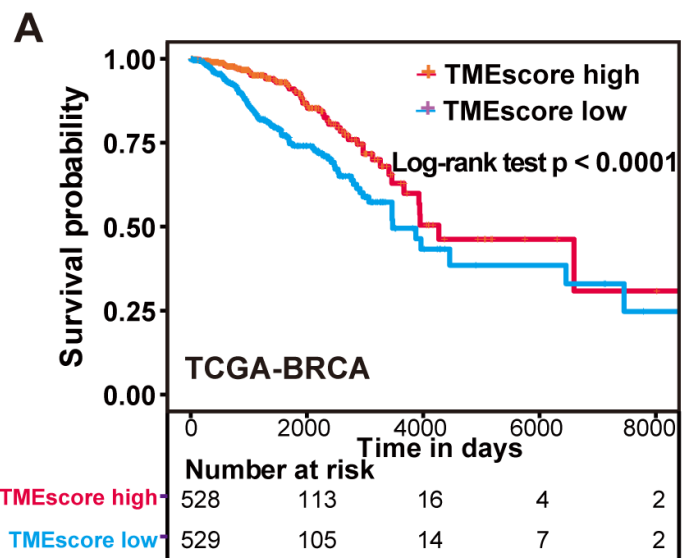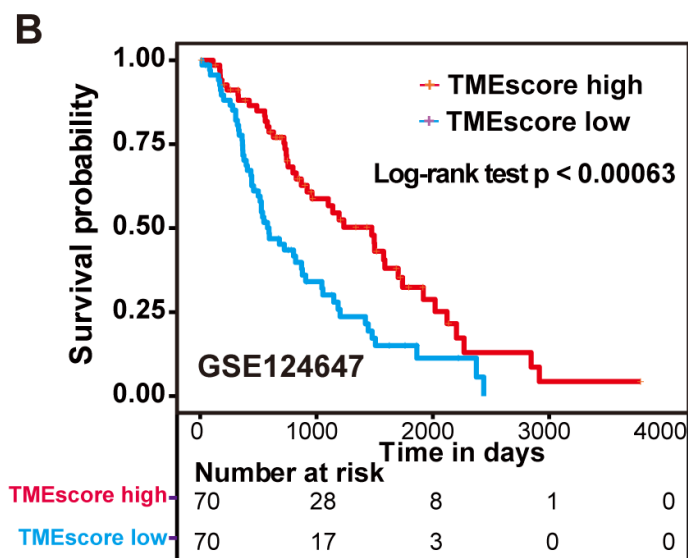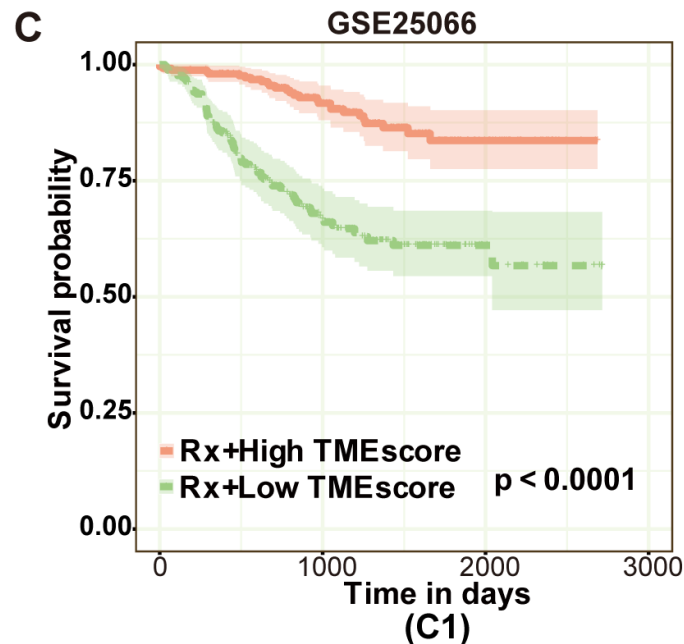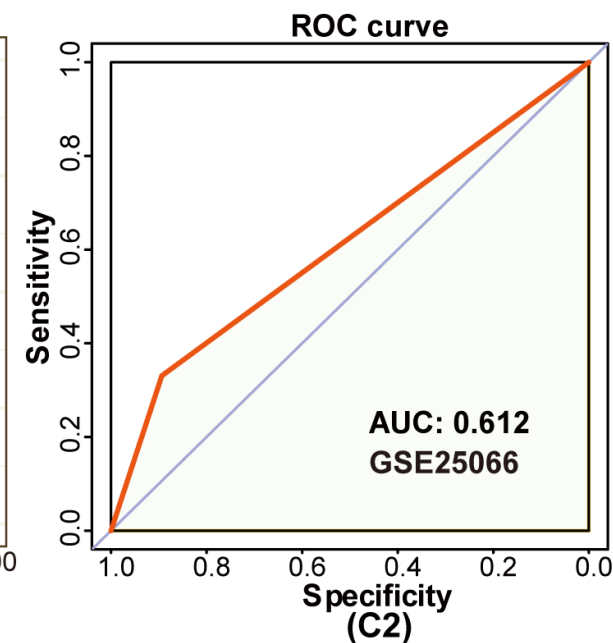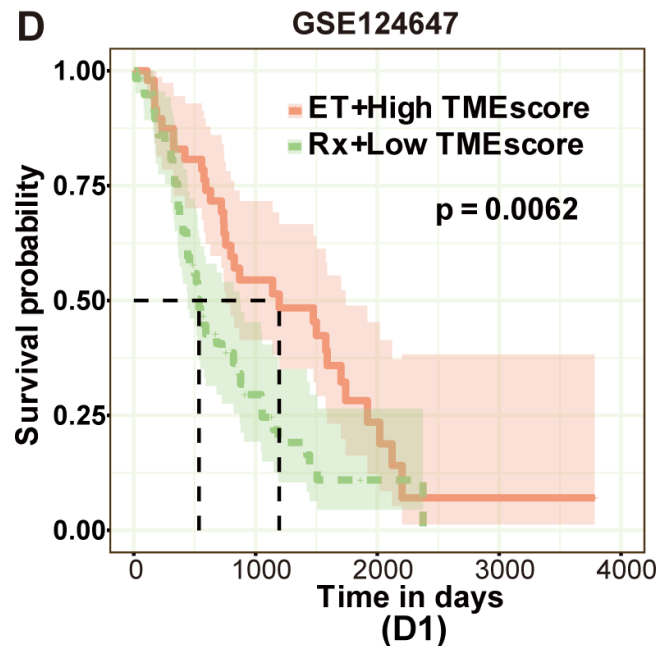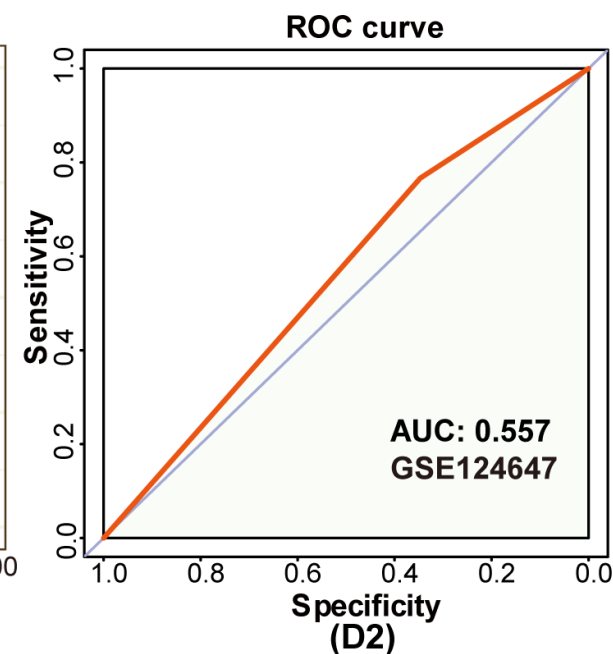

Supplement: Supplementary Materials — Supplementary Table 1: the basic characteristics of the 4 datasets: TCGA-BRCA, GSE96058, GSE124647, and GSE25066. Supplementary Figure 1: infiltrating cells in the TME. (A) The distribution ratio of 22 kinds of immune cells (B cells memory, dendritic cells activated, macrophages M0, etc.) in different samples: the ordinate represents the proportion of different immune cells, the abscissa represents different samples, and the color represents the type of immune cells. (B) The relationship between 22 kinds of immune cells and their relationship with survival (the larger the dot indicates the more related to survival, the thickness of the line indicates the strength of cell correlation). Supplementary Figure 2: the relationship between TMEscore and survival. (A) Consistent clustering results of differential genes: ConsensusClusterPlus was used for unsupervised class discovery (1000 iterations, k = 1 : 10). The optimal k value of 3 was determined using the elbow method and gap statics, combined with the correlation between the final classification and survival. The limma package of R was used to screen different types of differentially expressed genes (P < 0.05, |log2FC| > log2 (1.5)). 522 differentially expressed genes were obtained, and unsupervised cluster analysis was performed, and the samples were divided into three categories. (B) Functional enrichment analysis results of signature genes: functional enrichment analysis on 177 nonredundant genes using R ClusterProfiler revealed that this gene set was significantly enriched in immune-related pathways such as lymphocyte migration, lymphocyte chemotaxis, and leukocyte chemotaxis. (C) Survival analysis results of all enrolled samples based on TMEscore: a Cox regression model was used to determine the relationship between DEGs and the survival of samples. Next, genes were divided into 2 categories according to their coefficient values, and samples were divided into two groups based on high or low calculated TMEscores. [file 5673810.f1.zip › supplementary figures/SupplementaryFigure 3.pdf]
